# Supplementary material for: Cell-free biosynthesis combined with deep learning accelerates de novo-development of antimicrobial peptides
Source: Nat Commun. 2023 Nov 8;14:7197. doi: 10.1038/s41467-023-42434-9 (PMC10632401; doi:10.1038/s41467-023-42434-9)
Supplement: Supplementary file 1 — Supplementary Information [file 41467_2023_42434_MOESM1_ESM.pdf]

Supplementary information for

Cell-free biosynthesis combined with deep learning accelerates de novo-development of antimicrobial peptides

The supplementary information contains supplementary notes, tables, figures, and references:

Supplementary Notes 1-2 (p. 4-5)

Supplementary Note 1: Computational analysis of translation initiation rates.

Supplementary Note 2: Sequence similarity analyses using BLAST.

Supplementary Note 3: Molecular dynamics simulation of AMP-membrane interactions

Supplementary Tables 1-13 (p. 6-17)

Supplementary Table 1: Deep learning models training metrics.

Supplementary Table 2: Regressors classification metrics.

Supplementary Table 3: Our 30 functional AMPs (**Fig. 2**) predicted as AMP by other AMP prediction models.

Supplementary Table 4: Rounds of AMP generation, ranking, and testing with approaches and numbers.

Supplementary Table 5: Info of our 30 functional AMPs.

Supplementary Table 6: Frequent 3- and 4-mers in non-AMPs, generated, prioritized, and tested and function AMPs.

Supplementary Table 7: Blast results of 30 functional AMPs against UniProt.

Supplementary Table 8: Blast results of 30 functional AMPs against the AMPs training dataset.

Supplementary Table 9: Blast results of 30 functional AMPs against 500 tested AMPs.

Supplementary Table 10: MIC against *E. coli* and *B.subtilis* and HC50 (hemolysis) and CC50 (cytotoxicity) value of AMPs on human red blood cells and HCT116 human colon cells, respectively.

Supplementary Table 11: MIC (minimum inhibitory concentration), HC50 (hemolysis), and CC50 (cytotoxicity) values of BP100 and Cecropin B measured in this study.

Supplementary Table 12: Membrane equilibration scheme.

Supplementary Table 13: Human plasma membrane composition.

Supplementary Table 14: *E. coli* inner membrane composition.

Supplementary Table 15: TFA-based cleavage cocktails.

Supplementary Table 16: Columns for analytical and (semi-)preparative HPLC-MS.

Supplementary Figures 1-39 (p. 18-37)

Supplementary Fig. 1: Growth curves in Fig. 2b with error bars.

Supplementary Fig. 2: SDS-PAGE gel images of AMPs produced using cell-free protein synthesis (CFPS).

Supplementary Fig. 3: RBS calculator results on the expressibility of AMPs.

Supplementary Fig. 4: Properties of VAE models used in this study and the robustness analysis of VAE_2.

Supplementary Fig. 5: Physicochemical properties and amino acid composition of AMPs.

Supplementary Fig. 6: Complementary results on molecular dynamics simulations.

Supplementary Fig. 7: 21 days daily MIC measurements of the shortlisted AMPs for resistance study.

Supplementary Figs. 8-34: HPLC chromatogram of purified peptides.

Supplementary references (p. 38)

Supplementary Notes:

Supplementary Note 1: Computational analysis of translation initiation rates.

The bacterial killing potency of de novo AMP candidates that we screened via CFPS depends on their intrinsic antimicrobial activity (MIC, minimum inhibitory concentration), like natural AMPs, as well as their expressibility in the CFPS system. Since de novo-designed AMPs have very diverse sequences, their translation can be greatly affected by mRNA folding (**Supplementary Fig. 3a**). To examine the effect of mRNA sequences on translation, we used the RBS calculator[2] to predict the translation initiation rate (TIR) for each of the 500 tested AMPs. Despite AMPs being translated from the same RBS, the calculated TIR values are distributed in a wide range over four orders of magnitude (**Supplementary Fig. 3b**). Interestingly, the TIR values of the 30 functional AMPs are similarly distributed (**Supplementary Fig. 3b**) indicating that the translation initiation rate has not been a bottleneck in finding active AMPs. In addition to TIR which is calculated thermodynamically, the folding kinetic of mRNA also affects translation initiation such that the RBS calculator under-predicts slow folding mRNAs up to 10-fold[1]. Because mRNAs with low TIR fold slower (**Supplementary Fig. 3c**), their actual TIR value could be higher than the predicted one. This can narrow down the actual TIR range by an order of magnitude. Although we might have found more active AMPs if we had rationally designed RBS for all 500 tested AMPs, slow-translated functional peptides are more likely to have higher activity in killing bacteria in low amounts. These results show that cell-free production of AMPs enables the discovery of active AMPs despite their translation initiation rate, although the rational design of RBS for each AMP can lead to numerous functional AMPs.

Supplementary Note 2: Sequence similarity analyses using BLAST.
We sought to study the sequence similarities between our AMPs and both the training set and UniProt. For this purpose, we used the Basic Local Alignment Search Tool (BLAST) comparable to the previous works[3]. We assessed parameters including *E* (expected) value, percentage identity, query cover, and the raw alignment score. The *E* value is a parameter describing the number of hits that can be expected by chance in a dataset, also taking into account the length of a query. In BLAST searching for a query against the UniProt non-redundant database with ~240 million protein sequences, an *E* value ≤0.001 can refer to a significant match, hence homology, and the higher the *E* value, the higher the chance of coincidence. We performed BLAST for our 30 functional AMPs against the UniProt non-redundant database with an *E* value threshold of 10. For 25 AMPs we did not obtain any hit meaning that they had an *E* value >10. We saw a hit for only AMPs #6, #21, #23, #29, and #30 with *E* values of 7.1, 1.9, 9.7, 0.16, and 1.7, respectively (**Supplementary Table 7**). The BLAST hit with the lowest *E* value (0.16 for AMP #29 as the query) was a 511-amino acid bacterial outer membrane protein with an alignment score =40.5, percentage identity = 66.67%, and the query cover = 38%. Being a membrane protein and probably having a helical structure could be the reason for such a hit, nevertheless, the high *E* value and low percentage coverage in 38% of a peptide sequence do not indicate a homology. Additionally, none of the hits existed in the pretraining or training dataset. Next, we searched for sequence similarities between functional AMPs and the training dataset. Notably, inferring homology from the *E* value depends on the dataset size such that for the BLAST against our training dataset (~5000 sequences) the significance threshold would be around the *E* value of 10^{-7}.[3] We obtained no significant hit (**Supplementary Table 8**), with the highest *E* value being 0.004 for a search between AMP #21 (GIGKFQKMRFIGAIRASKGVAKGLLRIAAIRTGRRALTT)

and an AMP from the training dataset with the sequence of GILSTIKDFAIKAGKGAAKGLLEMASCKLSGQC. Moreover, BLAST searching of each functional AMP against all tested AMPs (including other functional ones) gave only one significant homology (**Supplementary Table 9**) meaning only one of the active AMPs had a similar VAE-generated sequence (not a functional AMP). Altogether, these results demonstrate that the AMPs we found in this work are unique and diverse.

Supplementary Note 3: Molecular dynamics simulation of AMP-membrane interactions.

All selected 30 AMPs are characterized by a high proportion of basic, aromatic and hydrophobic residues. AlphaFold[4] predicts most of them as α-helical, either as one long helix or two shorter helices connected by a disordered turn (**Fig. 2b**). The only three exceptions are AMPs #10 (disordered), #8 and #14 (containing β-strands). Together, the structural prediction and their sequences suggest that most of the AMPs act as amphipathic peptides that preferably insert into the membrane interface region of negatively charged membranes, such as the inner membrane (IM) of bacteria. We performed molecular dynamics (MD) simulation of AMPs near models of the IM and the human plasma membrane (PM) **(Fig. 3a**). In our MD simulations all AMPs bound much stronger to the IM than to the PM. This is well reflected by the distributions of the distances of the centers of mass of the peptides to the membrane midplane (**Fig. 3b**). The distributions are much narrower and closer to the membrane core for the IM simulations than for the PM simulations.

In the MD simulations, the AMPs bound the IM within at most 200 ns after being released near the membrane (**Supplementary Fig. 6a**) and did not unbind again. On the PM on the other hand, AMP binding was only ever observed transiently. In no case did an AMP insert into the PM deeper than into the IM. This applies not only at the centers of mass of the complete peptides, but also at the level of any individual atom (**Supplementary Fig. 6b**). The preference for IM over PM binding is unlikely to be due to the tighter packing of the PM alone, as even high lateral membrane tension did not result in PM binding as close and as strong as IM binding without any imposed lateral tension. In general, however, the lateral tension allows tighter binding to either membrane compared with the simulations without lateral tension. Yet, even with high lateral tension, no AMPs spontaneously traversed either membrane in any of our simulations.

Furthermore, all AMPs have a higher number of interactions with the IM than with the PM (**Supplementary Fig. 6c**) and on the IM many of these contacts are electrostatic interactions between the basic peptides and the acidic phospholipid headgroups. These electrostatic interactions with the lipid headgroups on top of possible hydrophobic interactions with the lipid tails may explain why IM binding was irreversible on the 1 µs timescale of the MD simulations, whereas PM binding exhibited frequent un- and rebinding.

In our MD simulations, we observed that most AMPs did not fully retain their predicted mostly α-helical structure, but became more disordered as time progressed (**Supplementary Fig. 6d**). This partial unfolding was more pronounced for the AMPs in the PM systems, where the peptides spent more of the simulated time in the bulk solvent rather than at the membrane interface. This hints towards a general mechanism where the AMPs are unfolded in solution and adopt an ordered structure when bound to the membrane. The amphipathic character of the structured state is stabilized by membrane interactions. Altogether, these results imply that (i) these peptides are most likely to act on membranes and (ii) they prefer bacterial over human membranes.

Supplementary Tables:

Supplementary Table 1: models training metrics.

Regressor	Negative data*	Positive data*	Accuracy		
CNN_MIC_regressor_v0	nonAMP[5] (5,582)	GRAMPA (5,102)	0.786		
CNN_MIC_regressor_v1	nonAMP[5] (5,582)	GRAMPA gram-specific 4,089 gram+, 4,619 gram-	0.835 gram- 0.887 gram+		
CNN_MIC_regressor_v2	UniProtKB nonAMP 10,612	GRAMPA gram-specific 4,089 gram+, 4,619 gram-	0.933 gram- 0.942 gram+		
RNN_MIC_regressor_v0	UniProtKB nonAMP 10,612	GRAMPA gram-specific 4,089 gram+, 4,619 gram-	0.942 gram- 0.949 gram+		
CNN_tox_classifier	17,434	8,992	0.942		
**Generator**	**Pretraining data**	**Training data**	**KL** term**	**KL loss**	**Recon. loss**
VAE_v0	-	GRAMPA (5,319)	-	1.179	3.331
VAE_v1	UniProtKB (~1.5 M)	GRAMPA (5,319)	-	1.265	3.027
VAE_v2	UniProtKB (~1.5 M)	GRAMPA (5,319)	+	2.494	4.925

*See Methods. ** Kullback-Leibner.

Supplementary Table 2: Classification metrics according to the rules in Witten & Witten[6] for our regressors and a few other AMP regressors. ACC: accuracy, SENS: sensitivity, SPEC: specificity, PPV: positive predictive value.

AMP prediction models	ACC	SENS	SPEC	PPV
Our RNN *E. coli*	94.2	87.2	94.3	87.2
Our RNN *S.aureus*	94.9	93.4	95.5	88.5
Our CNN *E. coli*	93.3	97.1	91.7	82.9
Our CNN *S. aureus*	94.2	96.0	94.3	87.2
CNN Witten & Witten[6]	96.2	97.8	97.0	97.8
AMP Scanner v2[7]	91.00	92.4	90.6	90.7
iAMP Pred[8]	79.7	86	73.4	76.5
CAMP-ANN[9]	88.9	85.2	92.6	92.0

Supplementary Table 3: Our 30 functional AMPs (**Fig. 2**) predicted as AMP by other AMP prediction models.

**AMP prediction models**	**Our AMPs (AMP #1-30) predicted as AMP by other models**	**Number of peptides (out of 30) predicted as AMP**
AMP Scanner v2[7]	1, 2, 3, 4, 5, 6, 7, 8, 9, 10, 11, 12, 13, 14,15, 16, 17, 18, 19, 20, 21, 22, 23, 24, 26, 28, 29, 30	28
iAMP Pred[8]	1, 2, 3, 5, 6, 7, 8, 9, 10, 11, 12, 13, 14, 15, 16, 17, 18, 19, 21, 22, 25, 26, 29, 30	24
DBAASP-SP[10]	1, 2, 3, 5, 9, 11, 12, 15, 16, 17, 21, 23, 24, 28	14
CAMP-ANN[9]	1, 2, 3, 6, 8, 9, 10, 11, 12, 13, 16, 18, 19, 20, 21, 23, 24, 29	18
CAMP-RF[9]	2, 3, 9, 10, 11, 18, 19, 20, 21, 26, 29	11
CAMP-SVM[9]	2, 3, 6, 8, 9, 10, 11, 13, 18, 19, 20, 21, 23, 29	14

Supplementary Table 4: Rounds of AMP generation, filtering, and prioritization with different models/approaches and numbers.

	**round 0**	**round 1**	**round 2**	**round 3**	**round 4**
**Generator**	VAE_v0	VAE_v0	VAE_v1	VAE_v1	VAE_v2
**Sampling**	Optimized Cecropin B*	random	random	random	random
**Regressor**	CNN reg_v0	CNN reg._v0	CNN reg._v1	CNN reg._v2 + Toxicity classifier	CNN reg._v2 + RNN reg._v0
**All generated peptides**	100	100,000	100,000	200,000	150,000
**Viable peptides**	100	9,220	9,117	18,218	29,457
**MIC-predicted and experimentally tested**	50	50	150	100	150
**Functional peptides discovered**	0	2	9	0	19
**Efficiency (%)**	**0**	**4**	**6**	**0**	**12.6**
**Top-ranked predicted AMPs (functional)****	50 (0)	0 (0)	50 (2)	50 (0)	50 (8)
**Random predicted AMPs (functional)****	0 (0)	50 (2)	100 (7)	50 (0)	100 (11)

For simplicity, in Fig. 2c we included the two functional AMPs from round 1 into the latent space of VAE_v1 together with functional AMPs of round 2. VAE_v0 had the same architecture and loss function as VAE_v1 however it was not pretrained. *****Gradient descent optimization at the neighborhood of Cecropin B in the latent space. **See Methods, section "Sampling and prioritizing of AMPs".

Supplementary Table 5: Our 30 functional AMPs with their sequences, generative and predictive models used for each, and specification of whether an AMP was randomly selected from those classified as AMP or was selected from the top-ranked (sorted, minimum MIC) candidates (See **Methods**).

AMP	Sequence	Generator	First Regressor	MIC, 1st Regressor	Second Regressor	MIC, 2nd Regressor	sorted/ random
AMP1	MLFGSRAKKYGKEAKQEKSFQYPKSS FVACKKKWKRSKHFFKTFKKKVS	VAE_v2	CNN reg_v2	0.184	RNN reg_v0	-0.881	sorted
AMP2	MWLKMRKCCGCGFKYCLKCVQKKGRI FKTLGKAKMWPKWFFKIGGKC	VAE_v2	CNN reg_v2	0.923	RNN reg_v0	-0.189	random
AMP3	MNNRGPLGRRFKARRKWKKFVAGKM KKKRKRFKGFKKKGGFTPFVKKFV	VAE_v2	CNN reg_v2	1.537	RNN reg_v0	1.808	random
AMP4	MEPFSKRFFLVFFKARCKCFFIFKSWF LFFFFFTFLVRRKKGVKQFFYE	VAE_v2	CNN reg_v2	0.784	RNN reg_v0	1.153	random
AMP5	MRRKTPVKWKTFFKALKHKKKIFKKTF EKFKFLAKGPAFLKGFDQKLKS	VAE_v2	CNN reg_v2	0.226	RNN reg_v0	-0.417	sorted
AMP6	MYLKKFLALKNSLKKLSPFKCAVKSWL KKCAEVTFYSKLLGRRGKKDGN	VAE_v2	CNN reg_v2	0.302	RNN reg_v0	1.855	random
AMP7	MRNFFKKTRLKYKGKKELIKKSRAFGL KTKKRSGFFFPRALKYEEEFY	VAE_v2	CNN reg_v2	1.166	RNN reg_v0	1.275	random
AMP8	MWFKWEWRPWFPSIFKKKYLAASNTE AKLCVKSKCYAKVGKGFAVILCF	VAE_v2	CNN reg_v2	0.504	RNN reg_v0	0.716	random
AMP9	MGWWFPPKTGNGAGKAFKKAAAAKW GGLFLKAAWFANKGEWGGGFPKGY	VAE_v2	CNN reg_v2	0.272	RNN reg_v0	1.406	random
AMP10	MCFCFKAGPKICRGLQRKKKKFKYQT SFTKTGFGFLTKPKSPAR	VAE_v2	CNN reg_v2	0.256	RNN reg_v0	-0.66	sorted
AMP11	MKKFIANGIGARACAKIGGFVKKGKMA KKKAAKAVKFVFKNGGKSKTKC	VAE_v2	CNN reg_v2	0.438	RNN reg_v0	-0.685	sorted
AMP12	MWFDRKKFFWPGVCLFLLFFPKRFYK KGPEKVFSFRKKYKFARKCKCKL	VAE_v2	CNN reg_v2	0.595	RNN reg_v0	-0.667	sorted
AMP13	MKQPSKTKTHFKYFLLFLKSVKKVAGF KKKKKKYHWRSYKEGSCFRKRT	VAE_v2	CNN reg_v2	1.447	RNN reg_v0	1.657	random
AMP14	MKEKKFFFFCFKKRRGFYKRRFFCKTT CFTYCFYKPRGTKTMPYVFSE	VAE_v2	CNN reg_v2	1.525	RNN reg_v0	0.212	random
AMP15	MEAFKKKPRLPLFKVKLTRFLERARGL GSYRIFEFFKKFGVKKFVSSLR	VAE_v2	CNN reg_v2	0.044	RNN reg_v0	-0.223	sorted
AMP16	MKAKKWFESLFKTFFKKGKGIYPKSSF EKEKKTDKKKKFGGWVWFKK	VAE_v2	CNN reg_v2	1.009	RNN reg_v0	0.439	random
AMP17	MWIKWKKPRKWGRRLKKKEKEELGD YIYLYCKVYRLFGFLPYFISKKTA	VAE_v2	CNN reg_v2	0.514	RNN reg_v0	0.077	random
AMP18	MCFFPRRKSKVKVKGGLCRLLFIFFKT TFCFKAKTKKEIKKGTGKKIVR	VAE_v2	CNN reg_v2	0.409	RNN reg_v0	-0.447	sorted
AMP19	MCFCRYRFFYRRRIRFFKWGPYFYVW FGFPFGRKAFFLSVFRRRFC	VAE_v2	CNN reg_v2	0.52	RNN reg_v0	-0.672	sorted

AMP20	MRGRPPKRIRSVIIAQTTTATAKKIVIVL LLIFSSSKRRR	VAE_v1	CNN reg_v1	0.76			random
AMP21	MGIGKFQKMRFIGAIRASKGVAKGLLRI AAIRTGRRALTT	VAE_v1	CNN reg_v1	0.872			random
AMP22	MGFSGILIKIALGIISISASISVVNTVIFFV VNGFGGVINFL	VAE_v1	CNN reg_v1	1.175			random
AMP23	MSTRSSSIRRLVEAVRTRFRAALRTVL FFALRTTKRRPRR	VAE_v1	CNN reg_v1	1.019			random
AMP24	MAIRIIGRLARVRARVVARVRSRLLADD PPEDLLRVARRRKGRRWLFLS	VAE_v1	CNN reg_v1	-0.285			sorted
AMP25	MATKTLFWWNNSCKNKTTIGIAVDAAL FGIIRSSFTAFY	VAE_v1	CNN reg_v1	1.784			random
AMP26	MKLRRRLRTRMVALLVLGVLFLLMLFFI IFLRRMLMRRFRA	VAE_v1	CNN reg_v1	-0.158			sorted
AMP27	MNTTSNMIHRAVQQKRISFRAAKLTVL FLFKRRLLRRLLRHHEN	VAE_v1	CNN reg_v1	1.08			random
AMP28	MMKIRNTLRSRKEAVRRIFSLRRRSVF TEMARAFRRFKAR	VAE_v1	CNN reg_v1	0.774			random
AMP29	MKKKKKGILKQNNKKKKYTLFNRMVVF LFLGFIMIIFVQKYKKVIYHK	VAE_v0	CNN reg_v0	-0.839			random
AMP30	MGFGLWGLFHFKMNVPNLFKNGFIFLII MIFTVWGLFFGKKKAYIEKFL	VAE_v0	CNN reg_v0	-0.805			random

Supplementary Table 6: Frequent 3- and 4-mers in non-AMPs, generated, prioritized and tested and function AMPs.

k-mers	UniProt nonAMP	Frequency	VAE training AMPs	Frequency	500 test AMPs	Frequency	30 functional AMPs	Frequency
3-mers	RRR	0.0046	LKK	0.0061	RRR	0.0125	KKK	0.0172
	KRT	0.0026	KKL	0.0053	KKK	0.0118	FKK	0.0097
	KGR	0.0025	LLK	0.0051	FKK	0.0056	KKF	0.0075
	GRK	0.0024	KLL	0.0045	FFF	0.0056	FFK	0.0067
	RKR	0.0024	KKI	0.0044	KKG	0.0042	KKG	0.006
	MKV	0.0022	AKK	0.0034	LLL	0.0032	LFL	0.0052
	RQG	0.0022	PRP	0.0031	RKK	0.003	FKA	0.0045
	VLA	0.0022	AGK	0.0031	GFK	0.0028	FFF	0.0045
	LAV	0.0022	LAK	0.003	FLF	0.0028	RRR	0.0045
	GFR	0.0022	AAK	0.003	KKF	0.0026	KKY	0.0037
	VIC	0.0022	IKK	0.0029	FFK	0.0026	GRR	0.0037
	GLL	0.0021	GLL	0.0028	RRL	0.0026	RRF	0.0037
4-mers	KQRQ	0.0019	KLLK	0.0022	RRRR	0.0055	KKKK	0.0061
	HGFR	0.0018	LKKL	0.002	KKKK	0.0041	FKKK	0.0038
	QRQG	0.0018	LLKK	0.0018	FFFF	0.0017	FKAR	0.0023
	HKQR	0.0018	KKLL	0.0018	FKKK	0.0015	KKFV	0.0023
	MKRT	0.0016	ASKV	0.0013	KKGF	0.0012	FFFF	0.0023
	MKVR	0.0015	RPRP	0.0012	KKKG	0.001	FFKK	0.0023
	RRRR	0.0014	KKKK	0.0012	FFKK	0.001	KKKY	0.0023
	KHKQ	0.0013	LKKI	0.0012	KQKK	0.001	FCFK	0.0023

Supplementary Table 7: NCBI BLASTP 2.13.0+ results of 30 functional AMPs against nonredundant UniProt containing 498M (498,091,743) sequences on August 5, 2022. The word size of 6, expect threshold of 10, PAM30 matrix, gap initiation penalty of 9 and gap extension penalty of 1, conditional compositional score matrix adjustment, and low complexity regions filter were used. The AMPs not shown here did not return any hit with an *E* value threshold of 10.

AMP	Hit reference ID	*E* value	Percent Identity	Query Cover	Max Score	Bit Score
AMP #6	MBW0556277.1	7.1	85.71%	28%	38.4	38.4
AMP #21	MCB5272487.1	1.9	61.54%	65%	39.2	39.2
AMP #23	REE03804.1	9.7	60.00%	55%	37.5	37.5
AMP #29	MBR6433148.1	0.16	59.26%	45%	43.5	43.5
AMP #30	ORE05204.1	1.7	66.67%	38%	40.5	40.5

Supplementary Table 8: NCBI BLASTP 2.13.0+ results of 30 functional AMPs against the training dataset containing ~5000 AMPs. The word size of 2, expect threshold of 10, BLOSUM62 matrix, gap initiation penalty of 11 and gap extension penalty of 1, and conditional compositional score matrix adjustment were used. The AMPs not shown here did not return any hit with an *E* value threshold of 10. Note that inferring homology from the *E* value depends on the dataset size such that for the BLAST against the training dataset the significance threshold would be around the *E* value of 10^{-7}.[3]

**AMP**	**Hit**	**Bit score**	***E* value**	**AMP**	**Hit**	**Bit score**	***E*-value**
AMP #1	train1855<br>train3986<br>train5028	16.9<br>16.9<br>16.9	7.7<br>8.4<br>8.8	AMP #16	train1491<br>train3557<br>train75<br>train2471<br>train5194<br>train3400<br>train1334	20.0<br>17.7<br>17.7<br>17.3<br>16.5<br>16.5<br>16.5	0.41<br>2.8<br>3.4<br>4.3<br>8.5<br>8.7<br>9.8
AMP #2	train68<br>train941<br>train3543<br>train939<br>train952<br>train579<br>train248	18.9<br>18.1<br>18.1<br>17.7<br>17.3<br>17.3<br>17.3	1.8<br>2.9<br>2.9<br>3.0<br>5.0<br>5.3<br>6.9	AMP #17	train471<br>train3918<br>train2948	18.5<br>18.1<br>16.9	1.6<br>2.7<br>7.5
AMP #3	train5167<br>train1437<br>train1426<br>train1425	17.3<br>16.9<br>16.9<br>16.9	4.4<br>8.2<br>8.6<br>9.1	AMP #18	train3793<br>train2453	17.3<br>16.9	6.2<br>7.8
AMP #5	train3915<br>train3957<br>train3528<br>train2105	18.1<br>16.9<br>16.5<br>16.9	2.4<br>5.7<br>8.2<br>8.5	AMP #19	train4740<br>train2912<br>train2509<br>train1446<br>train1447<br>train1315	19.6<br>19.6<br>18.1<br>17.7<br>17.7<br>16.9	0.70<br>0.74<br>2.6<br>4.9<br>5.0<br>9.0
AMP #6	train1503<br>train4288<br>train1185<br>train707	19.6<br>16.9<br>16.5<br>16.9	0.67<br>7.7<br>8.5<br>9.0	AMP #21	train1667<br>train1612<br>train1611<br>train2133<br>train2035<br>train2135<br>train2036	25.0<br>17.7<br>17.7<br>17.7<br>17.7<br>16.5<br>16.5	**0.004***<br>2.8<br>3.0<br>3.0<br>3.1<br>7.7<br>9.4
AMP #7	train4365	16.9	9.2	AMP #22	train529<br>train832<br>train1181<br>train833<br>train2604	18.1<br>17.3<br>16.5<br>16.5<br>16.2	2.5<br>4.0<br>7.3<br>7.7<br>9.8
AMP #8	train4630<br>train383<br>train2130	17.7<br>17.3<br>16.9	3.8<br>7.2<br>9.4	AMP #23	train1564<br>train1563	18.5<br>18.1	1.4<br>2.2

AMP #10	train3299<br>train746<br>train3300<br>train744<br>train4741<br>train543	22.3<br>19.6<br>18.5<br>17.7<br>17.3<br>16.5	0.06<br>0.49<br>2.4<br>3.2<br>7.9<br>8.3	AMP #24	train1932<br>train1931<br>train2063<br>train87<br>train4217	22.3<br>20.0<br>18.5<br>16.9<br>16.9	0.095<br>0.45<br>1.7<br>7.0<br>8.1
AMP #11	train3881	21.6	0.18	AMP #25	train4867<br>train2475<br>train2474<br>train469	18.5<br>17.7<br>17.7<br>17.3,	1.7<br>2.9<br>2.9<br>5.9
AMP #12	train2472	17.3	9.7	AMP #27	train1705<br>train4437	17.7<br>16.5	4.0<br>7.7
AMP #13	train1301<br>train4915<br>train1881	17.7<br>16.9<br>16.5	4.0<br>6.5<br>10.0	AMP #28	train1263	17.7	2.9
AMP #14	train9	18.1	2.9	AMP #29	train1368	16.9	7.6
AMP #15	train3476<br>train63<br>train1746<br>train3475	18.5<br>18.1<br>16.9<br>16.5	2.0<br>2.8<br>8.2<br>8.2	AMP #30	train1298<br>train831<br>train4893<br>train1297	19.2<br>18.1<br>17.7<br>16.9	1.1<br>2.6<br>4.9<br>8.0

***** Lowest E-value (not significant): AMP #21 (GIGKFQKMRFIGAIRASKGVAKGLLRIAAIRTGRRALTT) vs train1667 (GILSTIKDFAIKAGKGAAKGLLEMASCKLSGQC)

Supplementary Table 9: NCBI BLASTP 2.13.0+ results of 30 functional AMPs against 500 tested AMPs. The word size of 2, expect threshold of 10, BLOSUM62 matrix, gap initiation penalty of 11 and gap extension penalty of 1, and conditional compositional score matrix adjustment were used. Note that inferring homology from the *E* value depends on the dataset size such that for the BLAST against the training dataset the significance threshold would be around the *E* value of 10^{-8}.[3] All hits with an *E* value <10 are provided as a **Source Data** file.

***E* value**	**>10**	**1-10**	**10^{-3}-1**	**10^{-8}-0.10^{-3}**	**<0.10^{-8}**
Count	14782	175	41	1	1*

*The only significant *E*-value (1.00E-16): AMP #30 (GFGLWGLFHFKMNVPNLFKNGFIFLIIMIFTVWGLFFGKKKAYIEKFL) vs gen66 (GFGLWLLFQFKIRPPRLFKNGFLFLILMIFTTWILFFVKQKLFGMPFL)

Supplementary Table 10: MIC, HC50 and CC50 values (µM) of the plot in **Fig. 4a**. The values are the average of n=3 and n = 2 independent experiments for MIC and HC50/CC50 respectively.

	***E. coli* MIC**	***B. subtilis* MIC**	**CC50**	**HC50**
AMP #1	>100	0.8	113.0	>250
AMP #3	12.5	0.8	68.0	>250
AMP #5	2.1	0.5	68.4	82.9
AMP #6	25.0	2.1	67.5	73.8
AMP #7	>100	1.6	132.9	>250
AMP #9	25.0	3.1	146.0	>250
AMP #10	50.0	0.6	105.0	>250
AMP #12	37.5	6.3	>250	17.1
AMP #13	25.0	1.6	25.8	>250
AMP #14	37.5	6.3	>250	>250
AMP #15	25.0	0.8	30.7	24.8
AMP #16	6.3	0.8	39.3	180.2
AMP #17	12.5	6.3	133.7	>250
AMP #18	50.0	3.1	153.0	4.7
AMP #19	25.0	8.4	>250	>250
AMP #21	10.4	0.5	>250	>250
AMP #23	20.8	1.6	79.1	11.5
AMP #24	25.0	1.6	91.7	>250
AMP #26	100.0	37.5	>250	>250
AMP #27	12.5	0.4	75.7	105.1
AMP #28	25.0	0.4	145.0	>250
AMP #29	12.5	6.3	>250	50.6

Supplementary Table 11: MIC (minimum inhibitory concentration), HC50 (hemolysis), and CC50 (cytotoxicity) values of BP100 and Cecropin B measured in this study. The values are the average of n=3 independent experiments for *E. coli* and *B. subtilis* MIC and n = 2 independent experiments for others (n.d., not detected).

**MIC/HC50/CC50 (μM)**	**PB100**	**Cecropin B**
Escherichia coli MIC	3.1	0.4
Bacillus subtilis MIC	0.5	2.6
Acinetobacter baumannii MIC	0.6	0.2
Enterobacter cloacae MIC	12.5	0.4
Klebsiella pneumoniae MIC	2.4	0.2
Pseudomonas aeruginosa MIC	6.3	3.1
Staphylococcus aureus (MRSA) MIC	6.3	>25
Enterococcus faecium MIC	4.7	>25
Hemolysis (HC50)	96.0	nd
Cytotoxicity (CC50)	56.8	154.2

Supplementary Table 12: Membrane equilibration scheme.

Step	Time[ns]	Timestep [fs]	Ensemble	Headgroup position restraints [kJ mol^{-1}]	Tail dihedral angle restraints [kJ mol^{-1}]
**EM**				1000	1000
**1**	1.25	1	NVT	1000	1000
**2**	1.25	1	NVT	400	400
**3**	1.25	1	NPT	400	200
**4**	0.5	2	NPT	200	200
**5**	0.5	2	NPT	40	100
**6**	0.5	2	NPT	0	0

Supplementary Table 13: Human plasma membrane composition.

Lipid	Full name	Abundance [%]
CHOL	Cholesterol	35.8
PSM	N-palmitoyl-D-erythro-sphingosylphosphorylcholine	13.1
NSM	N-nervonoyl-D-oleoyl-sphingosylphosphorylcholine	10.0
LSM	N-lignoceroyl-D-oleoyl-sphingosylphosphorylcholine	8.4
PLPC	1-palmitoyl-2-linoleoyl-sn-glycero-3-phosphatidylcholine	16.2
SOPC	1-stearoyl-2-oleoylphosphatidylcholine	7.5
PAPC	1-palmitoyl-2-arachidonoyl-glycero-3-phosphatidylcholine	5.6
PLA20(PE)	1-O-stearoyl-2-O-arachidonoyl-glycero-3-phosphatidylethanolamine	2.2
SAPS	1-stearoyl-2-arachidonoyl-glycero-3-phosphatidylserine	1.2

Supplementary Table 14: *E. coli* inner membrane composition.

Lipid	Full name	Abundance [%]
PVPE	1-palmitoyl-2-vacenoyl-sn-glycero-3-phosphatidylethanolamine	75
PVPG	1-palmitoyl-2-vacenoyl-sn-glycero-3-phosphatidylglycerol	20
PVCL2	1-palmitoyl-2-vacenoyl-cardiolipin	5

Supplementary Table 15: TFA-based cleavage cocktails. Depending on the content of the oxidation prone amino acids Cys, Met and Trp one of the following cleavage cocktails has been used.

Cocktail	Cocktail Composition (v/v)
Cleavage Cocktail A	82.5% TFA, 5.0% H_2O, 5.0% phenol, 5.0% thioanisole, 2.5 % EDT
Cleavage Cocktail B	90 % TFA, 4.0% TMSBr, 4.0% thioanisole, 2.0% EDT

Supplementary Table 16: Columns for analytical and (semi-)preparative HPLC-MS. Column 1 was used for the preparative purification of peptide crude while column 2 was for the characterization of the final purified peptides.

Column	Type	Dimensions	Flow	Purpose
Column 1	XBridge Prep C18 OBD (Waters)	250 x 19 mm, 5 µm	16 mL/min	purification
Column 2	Eclipse XBD-C18 (Agilent Technologies)	150 x 4.6 mm, 5 µm	1 mL/min	analysis

Supplementary Figures:

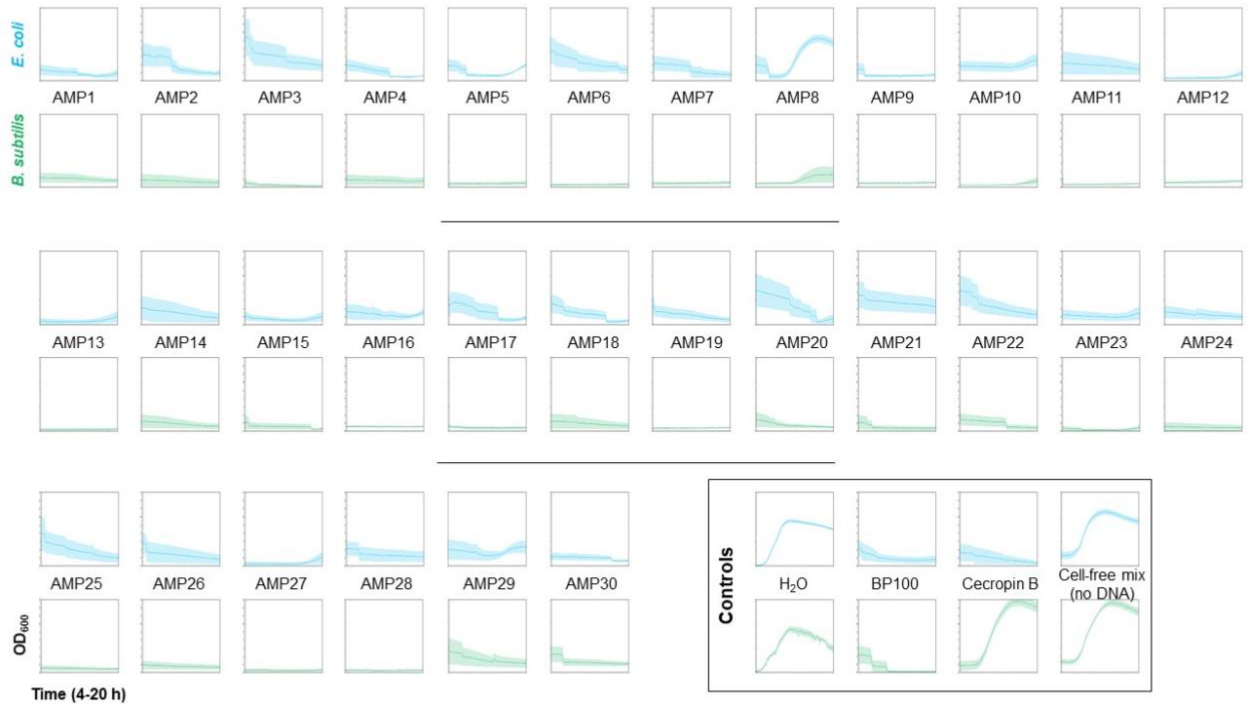

Supplementary Fig. 1: OD_{600} over time 4-20 h growth curves (n=3 independent experiments) in **Fig. 2b** with error bars as standard deviation. Raw data is provided as a **Source Data** file.

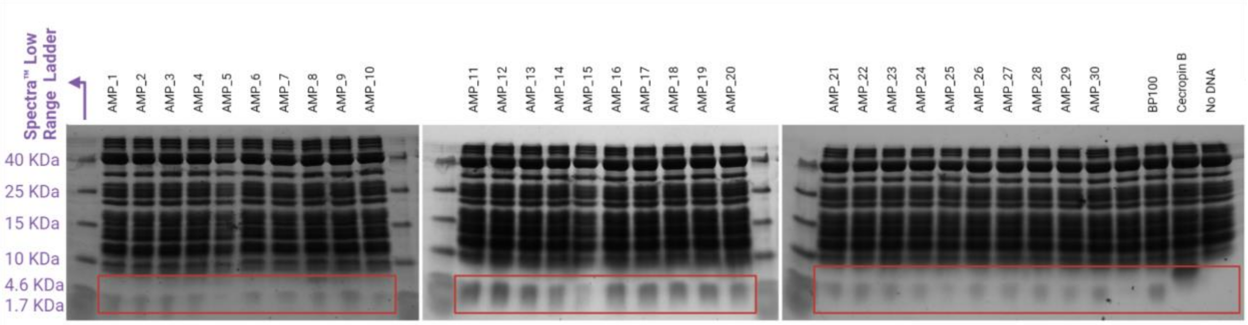

Supplementary Fig. 2: SDS-PAGE of AMPs produced using cell-free protein synthesis. This experiment was performed with no replicates and the raw images are provided as a **Source Data** file. Created with BioRender.com.

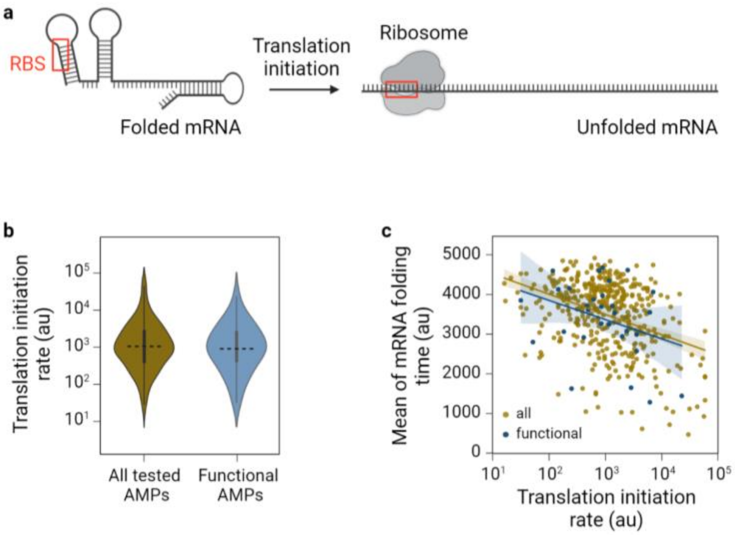

Supplementary Fig. 3: RBS calculator simulations on the translation of AMPs. **a,** Schematic of the translation initiation by mRNA unfolding and binding the ribosome to the RBS (ribosome binding site). **b,** The translation initiation rate (TIR) calculated using the RBS calculator for all 500 tested AMPs (yellow) and 30 functional AMPs (blue). The dashed lines show the median. **c,** Mean of mRNA folding time versus translation initiation rate for all 500 tested AMPs (yellow) and 30 functional AMPs (blue). RBS Calculator v1.0[2] was used. For folding times, we used Kinfold[11] run 1000 times for each sequence, with a folding time cut-off of 5000 and the rest of the parameters as default, EnergyModel: dangle=2 Temp=37.0 logML=logarithmic Par=VRNA-1.4, MoveSet: noShift=off noLP=off, Simulation: num=1000 time=5000.00 seed=clock fpt=on mc=Kawasaki, Simulation: phi=1 pbounds=0.1 0.1 2. au: arbitrary unit. Created with BioRender.com.

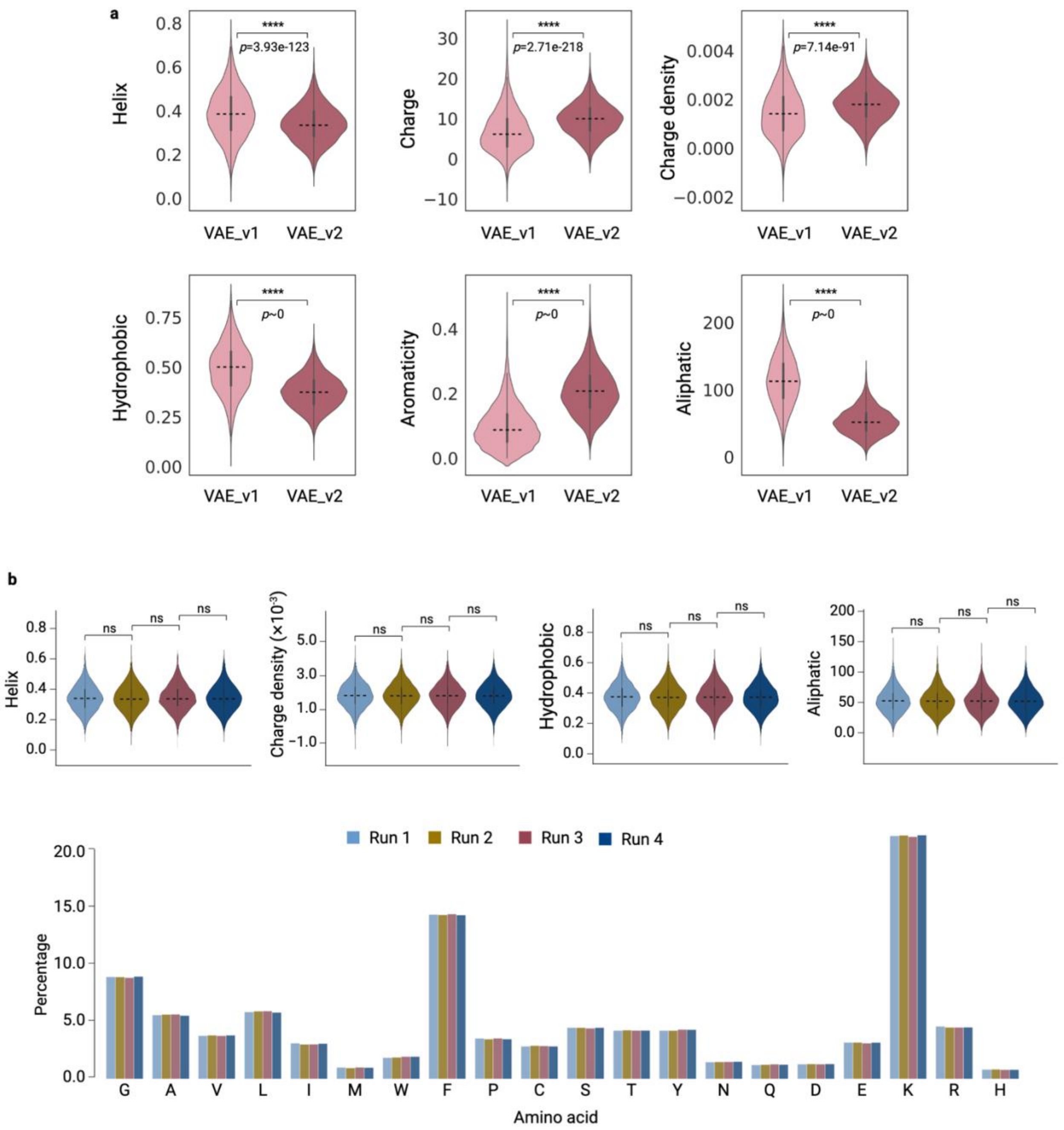

Supplementary Fig. 4: Properties of VAE (variational autoencoder) models used in this study with n=5000 generated peptide for each (**a**) and the robustness analysis of VAE_2 with four independent runs of n=5000 peptide generation showing no significant difference in the generated peptides for physicochemical properties and amino acids composition (**b**). These features were calculated on viable peptides with 36-48 amino acids generated using each VAE. These features were computed using Biopython 1.79[12] and the modlAMP 4.3.0[13] packages. Mann Whitney test with Bonferroni adjustment was used, ns: not significant (0.05 < p < 1), ****: p <= 0.0001. Source data for this figure are provided (see Data Availability). Created with BioRender.com.

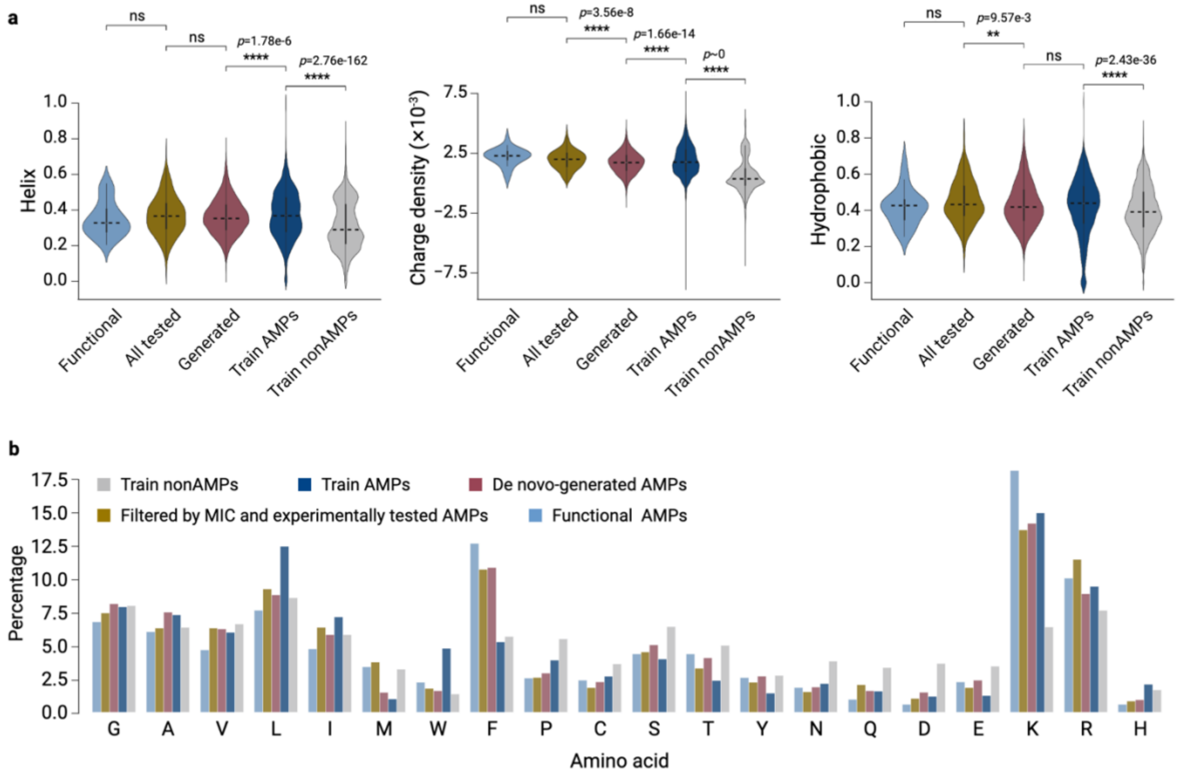

Supplementary Fig. 5: Physicochemical properties (**a**) and amino acid composition of AMPs (**b**) computed using Biopython 1.79[12] and the modlAMP 4.3.0[13] packages. Number of datapoints are as follows; functional AMPs n=30, tested AMPs n=450, generated AMPs (both VAEs) n=10,000, train AMPs n=5,319, train nonAMPs n=10,602. Mann Whitney test with Bonferroni adjustment was used, ns: not significant (0.05 < *p* < 1), **: 0.001 < *p* < 0.01, ****: *p* <= 0.0001. Source data for this figure are provided (see Data Availability). Created with BioRender.com.

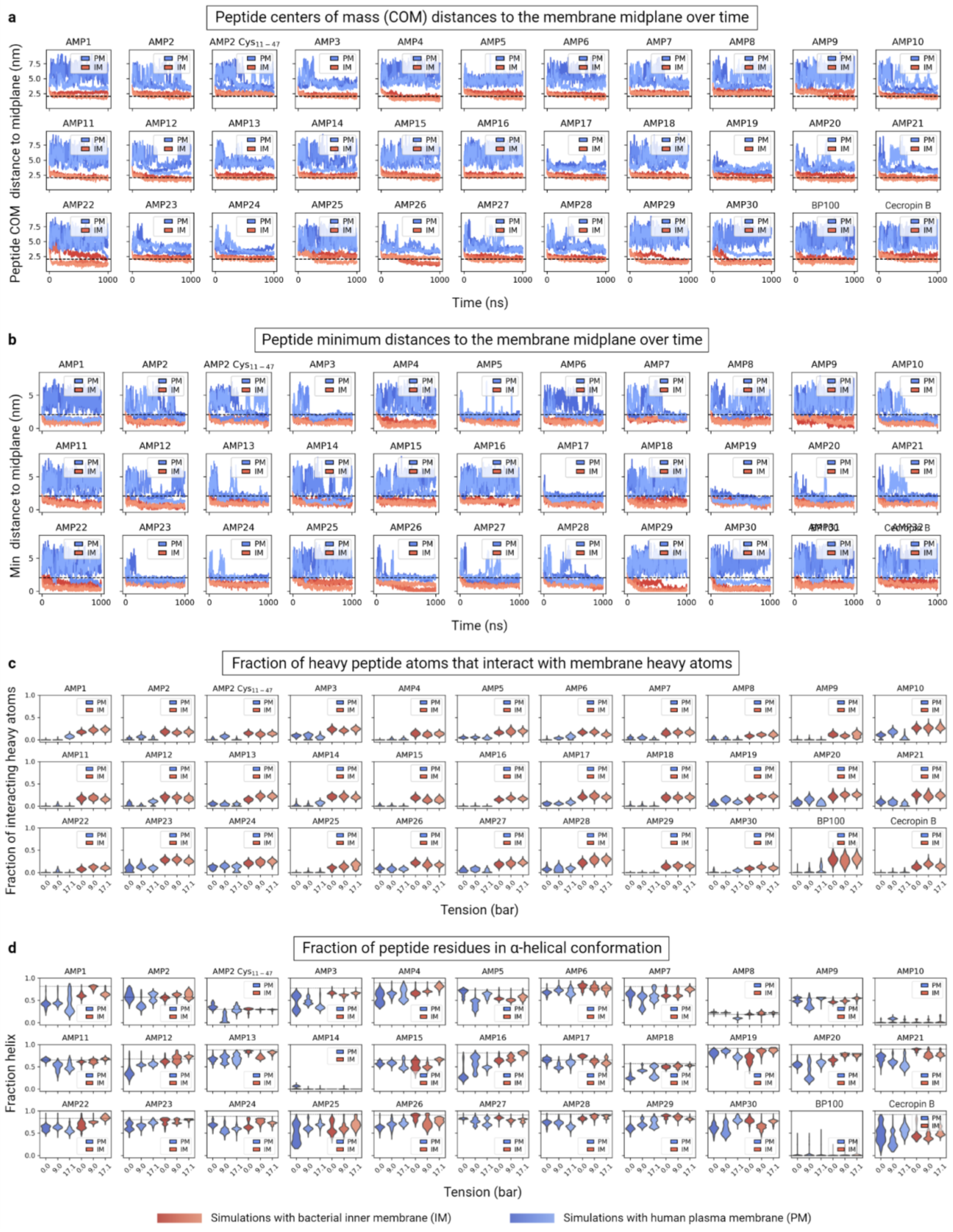

Supplementary Fig. 6: Complementary results on molecular dynamics simulations. **a,** Distances along the direction of the membrane normal between the AMP centers of mass and the membrane midplane plotted vs. time for each replicate, run with different lateral tensions (darkest: 0 bar;

lighter: 9 bar; lightest: 17.1 bar) and with different membranes (blue: PM; red: IM). The dotted line indicates the headgroup phosphate positions. **b,** Minimum distances along the direction of the membrane normal between any heavy AMP atom and the membrane midplane plotted vs. time for each replicate run with different lateral tensions (darkest: 0 bar; lighter: 9 bar; lightest: 17.1 bar) and with different membranes (blue: PM; red: IM). The dotted line indicates the headgroup phosphate positions. **c,** Distributions of the fractions of heavy peptide atoms that interact with membrane heavy atoms (cutoff 3.5 Å). Distributions are calculated from the last 950 ns of 1 µs long replicates, run with different lateral tensions and with different membranes (blue: PM; red: IM). **d,** Distributions of the fractions of AMP residues that are in α-helical conformation. Distributions are calculated from the last 950 ns of 1 µs long replicates, run with different lateral tensions and with different membranes (blue: PM; red: IM). The dotted line indicates the helicity in the initial model prediction by AlphaFold[4]. Created with BioRender.com.

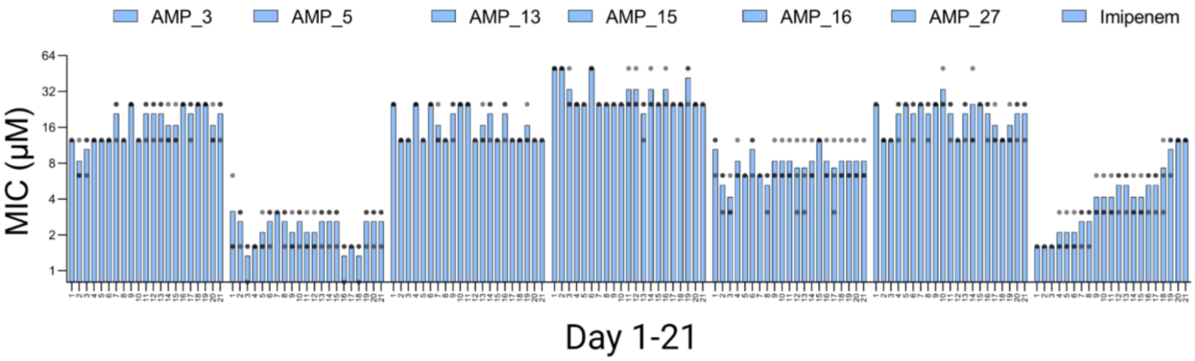

Supplementary Fig. 7. 21 days daily MIC measurements of the six broad-band AMPs for resistance study. The y axis is in log2 scale. See also **Fig 6a**. Bars are the average of n = 3 independent experiments. Source data for this figure are provided as a **Source Data** file. Created with BioRender.com.

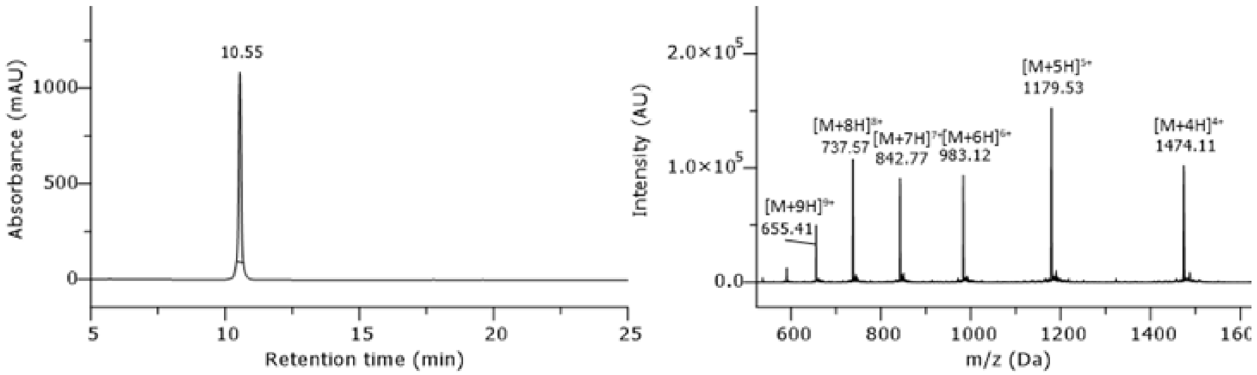

Supplementary Fig. 8: HPLC chromatogram of purified peptide AMP #1. Gradient 5-95% B in column 2, monitored at 220 nm. Absorbance mAU: milli-absorbance unit. Intensity AU: arbitrary unit.

H_2N-MLFGSRAKKYGKEAKQEKSFQYPKSSFVACKKKWKRSKHFFKTFKKKVS-$CONH_2$; peptide has been synthesized in 5 µmol scale. After purification (column 1, 05-50% MeCN), the 19 x TFA salt product (9.40 mg, 1.17 µmol, 23%) was obtained as a white solid. t_R = 10.55 min. Purity ≥ 99%. Formula: $C_{275}H_{434}N_{76}O_{64}S_2$. Molecular weight: 5892.98 g/mol. HRMS-ESI+ (m/z): $[M+10H]^{10+}$ calcd.: 590.2329; found: 590.2328.

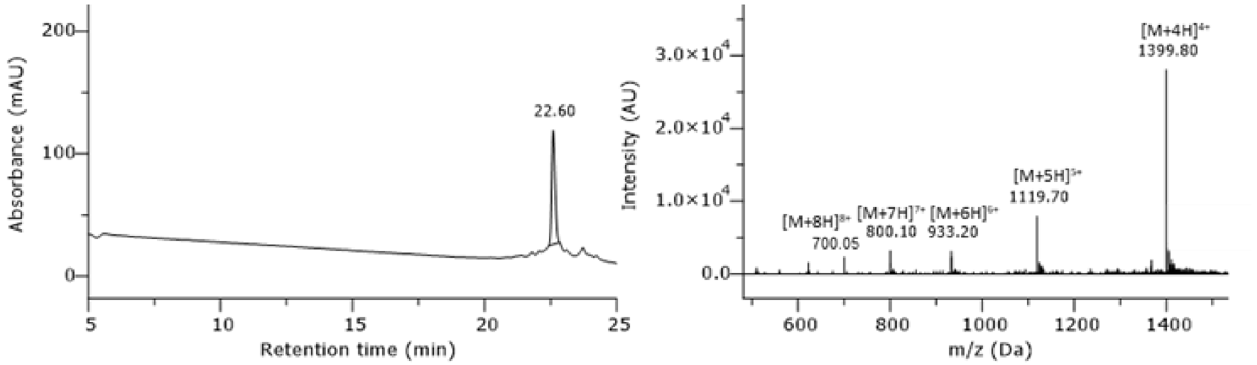

Supplementary Fig. 9: HPLC chromatogram of purified peptide AMP #2. Gradient 15-35% B, with addition of 1 mM TCEP, in column 2, monitored at 220 nm. Absorbance mAU: milli-absorbance unit. Intensity AU: arbitrary unit.

H_2N-MWLKMRKCCGCGFKYCLKCVQKKGRIFKTLGKAKMWPKWFFKIGGKC-$CONH_2$; peptide has been synthesized in 5 µmol scale. After purification (column 1, 05-50% MeCN), the 15 x TFA salt product (3.49 mg, 0.47 µmol, 9%) was obtained as a white solid. t_R = 22.60 min. Purity > 83%. Formula: $C_{259}H_{412}N_{70}O_{50}S_9$. Molecular weight: 5595.06 g/mol. HRMS-ESI+ (m/z): $[M+9H]^{9+}$ calcd.: 622.5562; found: 622.5576.

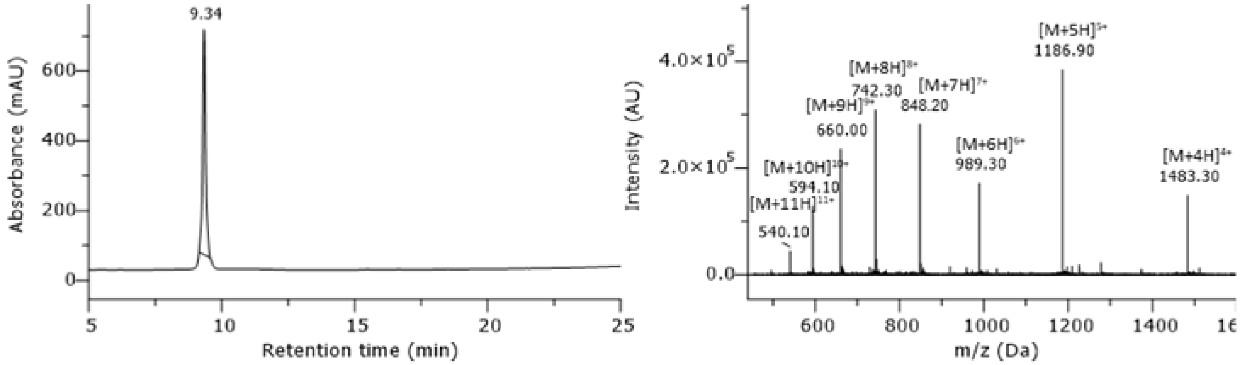

Supplementary Fig. 10: HPLC chromatogram of purified peptide AMP #3. Gradient 5-95% B in column 2, monitored at 220 nm. Absorbance mAU: milli-absorbance unit. Intensity AU: arbitrary unit.

H_2N-MNNRGPLGRRFKARRKWKKFVAGKMKKKRKRFKGFKKKGGFTPFVKKFV-$CONH_2$; peptide has been synthesized in 5 µmol scale. After purification (column 1, 05-35% MeCN), the 23 x TFA salt product (9.60 mg, 1.12 µmol, 22%) was obtained as a white solid. t_R = 9.34 min. Purity ≥ 99%. Formula: $C_{277}H_{457}N_{89}O_{52}S_2$. Molecular weight: 5930.28 g/mol. HRMS-ESI+ (m/z): $[M+7H]^{7+}$ calcd.: 848.0840; found: 848.0827.

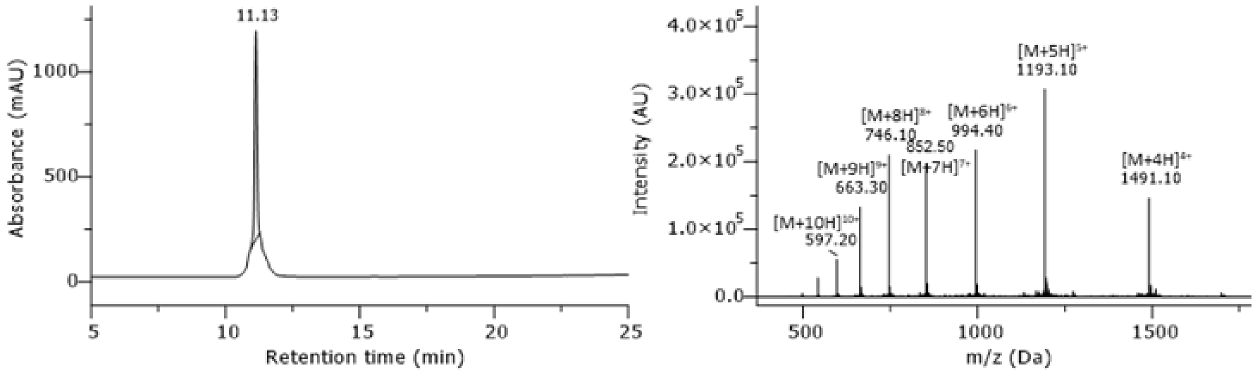

Supplementary Fig. 11: HPLC chromatogram of purified peptide AMP #5. Gradient 5-95% B in column 2, monitored at 220 nm. Absorbance mAU: milli-absorbance unit. Intensity AU: arbitrary unit.

H_2N-MRRKTPVKWKTFFKALKHKKKIFKKTFEKFKFLAKGPAFLKGFDQKLKS-$CONH_2$; peptide has been synthesized in 5 µmol scale. After purification (column 1, 05-50% MeCN), the 20 x TFA salt product (9.27 mg, 1.12 µmol, 22%) was obtained as a white solid. t_R = 11.13 min. Approximate purity > 90%. Formula: $C_{289}H_{462}N_{76}O_{58}S$. Molecular weight: 5961.29 g/mol. HRMS-ESI+ (m/z): $[M+7H]^{7+}$ calcd.: 852.5122; found: 852.5127.

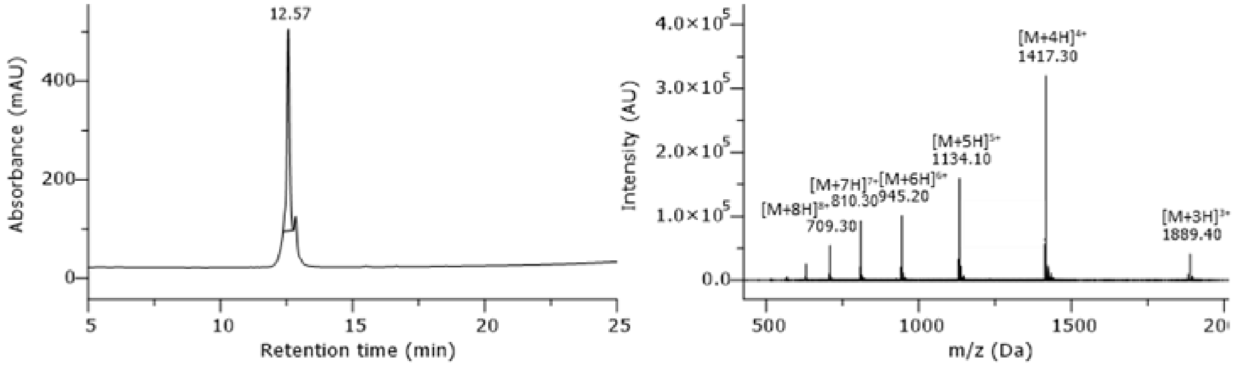

Supplementary Fig. 12: HPLC chromatogram of purified peptide AMP #6. Gradient 5-95% B in column 2, monitored at 220 nm. Absorbance mAU: milli-absorbance unit. Intensity AU: arbitrary unit.

H_2N-MYLKKFLALKNSLKKLSPFKCAVKSWLKKCAEVTFYSKLLGRRGKKDGN-$CONH_2$; peptide has been synthesized in 5 µmol scale. After purification (column 1, 05-50% MeCN), the 15 x TFA salt product (4.67 mg, 0.63 µmol, 13 %) was obtained as a white solid. t_R = 12.57 min. Approximate purity > 90%. Formula: $C_{262}H_{433}N_{71}O_{62}S_3$. Molecular weight: 5665.87 g/mol. HRMS-ESI+ (m/z): $[M+6H]^{6+}$ calcd.: 945.2098; found: 945.2128.

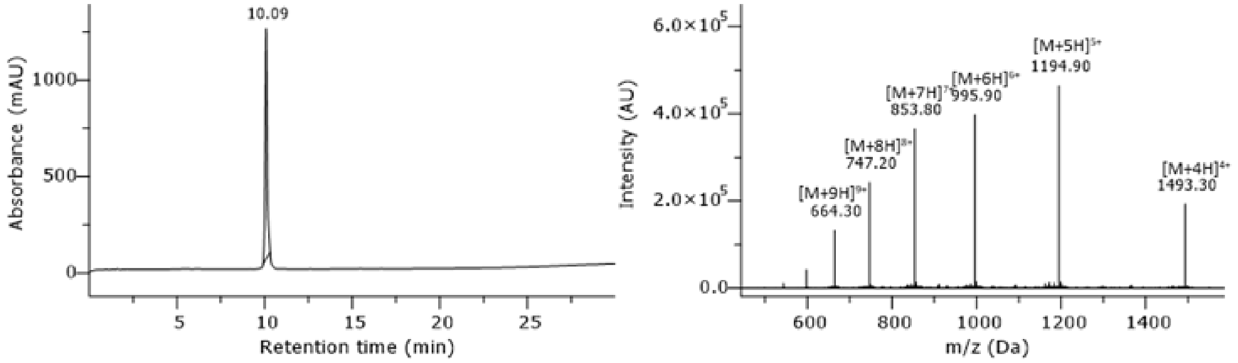

Supplementary Fig. 13: HPLC chromatogram of purified peptide AMP #7. Gradient 5-95% B in column 2, monitored at 220 nm. Absorbance mAU: milli-absorbance unit. Intensity AU: arbitrary unit.

H_2N-MRNFFKKTRLKYKGKKELIKKSRAFGLKTKKRSGFFFPRALKYEEEFY-$CONH_2$; peptide has been synthesized in 5 µmol scale. After purification (column 1, 05-50% MeCN), the 18 x TFA salt product (3.49 mg, 0.44 µmol, 9%) was obtained as a white solid. t_R = 10.09 min. Purity ≥ 99%. Formula: $C_{282}H_{445}N_{77}O_{64}S$. Molecular weight: 5970.09 g/mol. HRMS-ESI+ (m/z): $[M+6H]^{6+}$ calcd.: 995.9029; found: 995.9010.

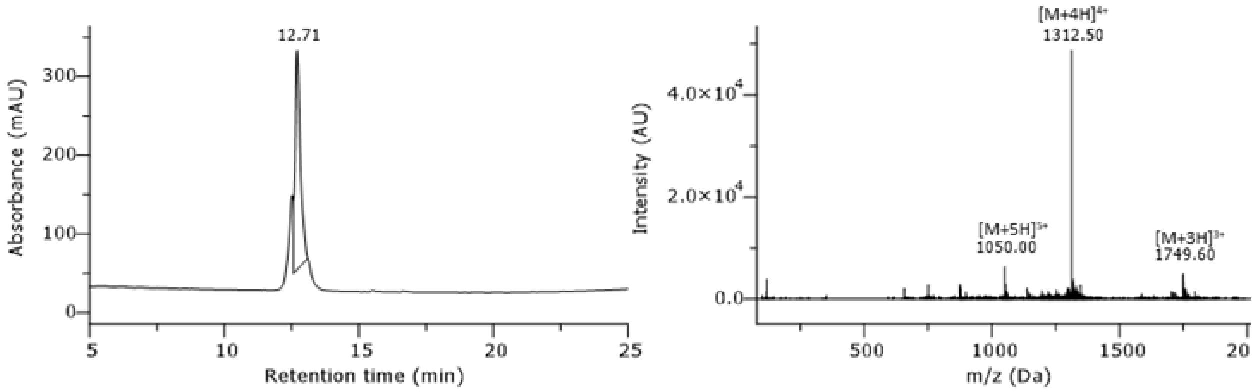

Supplementary Fig. 14: HPLC chromatogram of purified peptide AMP #9. Gradient 5-95% B in column 2, monitored at 220 nm. Absorbance mAU: milli-absorbance unit. Intensity AU: arbitrary unit.

H_2N-MGWWFPPKTGNGAGKAFKKAAAAKWGGLFLKAAWFANKGEWGGGFPKGY-$CONH_2$; peptide has been synthesized in 5 µmol scale. After purification (column 1, 05-50% MeCN), the 9 x TFA salt product (5.27 mg, 0.84 µmol, 17 %) was obtained as a white solid. t_R = 12.71 min. Approximate purity 75%. Formula: $C_{255}H_{359}N_{65}O_{55}S$. Molecular weight: 5247.04 g/mol. HRMS-ESI+ (m/z): $[M+6H]^{6+}$ calcd.: 875.4589; found: 875.4609.

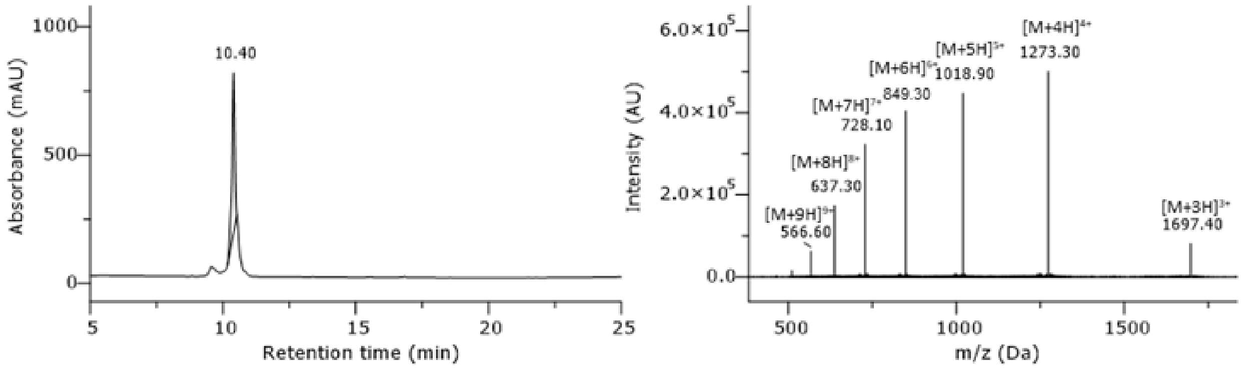

Supplementary Fig. 15: HPLC chromatogram of purified peptide AMP #10. Gradient 5-95% B in column 2, monitored at 220 nm. Absorbance mAU: milli-absorbance unit. Intensity AU: arbitrary unit.

H_2N-MCFCFKAGPKICRGLQRKKKKFKYQTSFTKTGFGFLTKPKSPAR-$CONH_2$; peptide has been synthesized in 5 µmol scale. After purification (column 1, 05-50% MeCN), the 14 x TFA salt product (2.98 mg, 0.45 µmol, 9%) was obtained as a white solid. t_R = 10.40 min. Purity 93%. Formula: $C_{234}H_{376}N_{66}O_{53}S_4$. Molecular weight: 5090.15 g/mol. HRMS-ESI+ (m/z): $[M+6H]^{6+}$ calcd.: 849.3025; found: 849.3021.

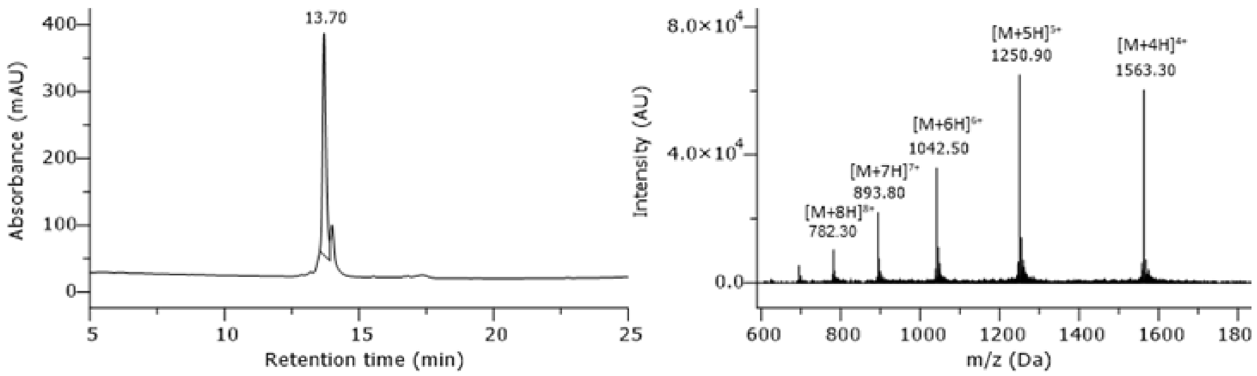

Supplementary Fig. 16: HPLC chromatogram of purified peptide AMP #12. Gradient 5-95% B in column 2, monitored at 220 nm. Absorbance mAU: milli-absorbance unit. Intensity AU: arbitrary unit.

H_2N-MWFDRKKFFWPGVCLFLLFFPKRFYKKGPEKVFSFRKKYKFARKCKCKL-$CONH_2$; peptide has been synthesized in 5 µmol scale. After purification (column 1, 05-75% MeCN), the 17 x TFA salt product (3.64 mg, 0.44 µmol, 9%) was obtained as a white solid. t_R = 13.70 min. Approximate purity 86%. Formula: $C_{308}H_{458}N_{76}O_{56}S_4$. Molecular weight: 6249.66 g/mol. HRMS-ESI+ (m/z): $[M+9H]^{9+}$ calcd.: 695.3885; found: 695.3935.

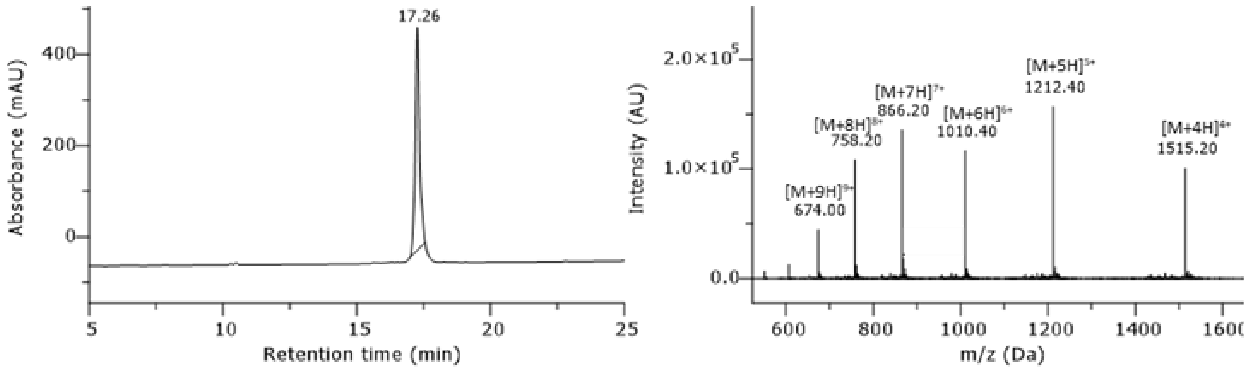

Supplementary Fig. 17: HPLC chromatogram of purified peptide AMP #13. Gradient 5-50% B, with addition of 1 mM TCEP, in column 2, monitored at 220 nm. Absorbance mAU: milli-absorbance unit. Intensity AU: arbitrary unit.

H_2N-MKQPSKTKTHFKYFLLFLKSVKKVAGFKKKKKKYHWRSYKEGSCFRKRT-$CONH_2$; peptide has been synthesized in 5 µmol scale. After purification (column 1, 05-50% MeCN), the 21 x TFA salt product (2.65 mg, 0.31 µmol, 6%) was obtained as a white solid. t_R = 17.26 min. Purity ≥ 99%. Formula: $C_{285}H_{454}N_{80}O_{62}S_2$. Molecular weight: 6057.28 g/mol. HRMS-ESI+ (m/z): $[M+7H]^{7+}$ calcd.: 866.2165; found: 866.2123.

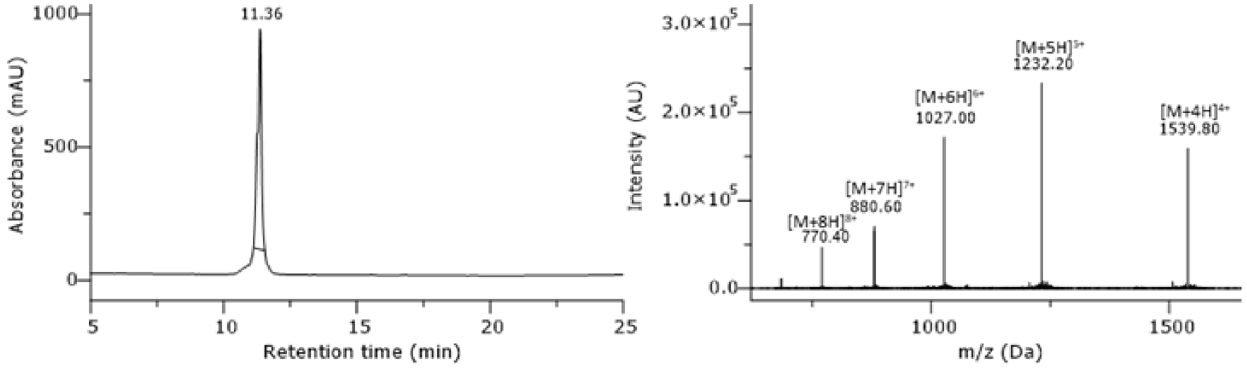

Supplementary Fig. 18: HPLC chromatogram of purified peptide AMP #14. Gradient 5-95% B in column 2, monitored at 220 nm. Absorbance mAU: milli-absorbance unit. Intensity AU: arbitrary unit.

H_2N-MKEKKFFFFCFKKRRGFYKRRFFCKTTCFTYCFYKPRGTKTMPYVFSE-$CONH_2$; peptide has been synthesized in 5 µmol scale. After purification (column 1, 05-50% MeCN), the 15 x TFA salt product (3.24 mg, 0.41 µmol, 8%) was obtained as a white solid. t_R = 11.36 min. Approximate purity ≥ 89%. Formula: $C_{293}H_{427}N_{73}O_{62}S_6$. Molecular weight: 6156.36 g/mol. HRMS-ESI+ (m/z): $[M+7H]^{7+}$ calcd.: 880.5921; found: 880.5912.

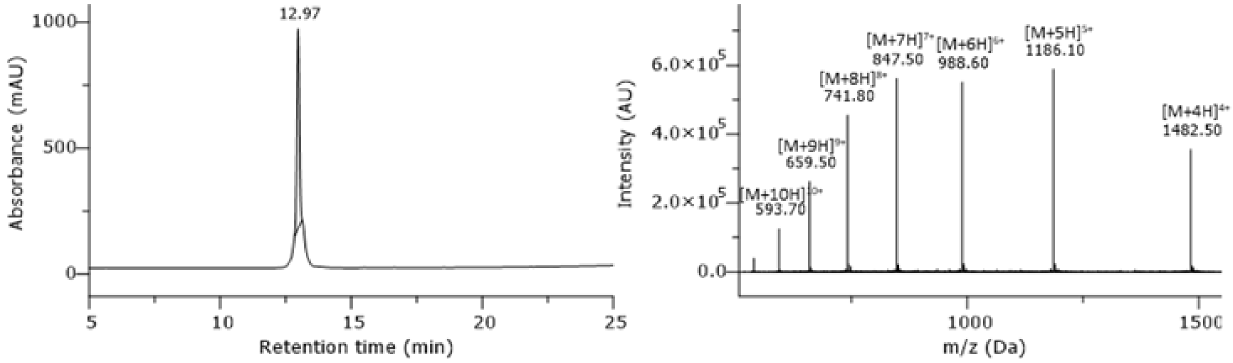

Supplementary Fig. 19: HPLC chromatogram of purified peptide AMP #15. Gradient 5-95% B in column 2, monitored at 220 nm. Absorbance mAU: milli-absorbance unit. Intensity AU: arbitrary unit.

H_2N-MEAFKKKPRLPLFKVKLTRFLERARGLGSYRIFEFFKKFGVKKFVSSLR-$CONH_2$; peptide has been synthesized in 5 µmol scale. After purification (column 1, 05-50% MeCN), the 16 x TFA salt product (3.69 mg, 0.48 µmol, 10 %) was obtained as a white solid. t_R = 12.97 min. Purity ≥ 97%. Formula: $C_{283}H_{453}N_{77}O_{60}S$. Molecular weight: 5926.16 g/mol. HRMS-ESI+ (m/z): $[M+6H]^{6+}$ calcd.: 988.5834; found: 988.5846.

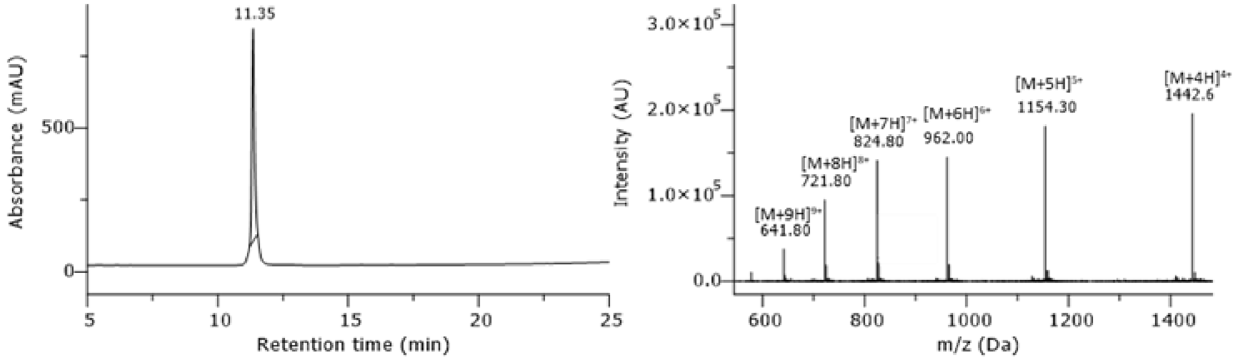

Supplementary Fig. 20: HPLC chromatogram of purified peptide AMP #16. Gradient 5-95% B in column 2, monitored at 220 nm. Absorbance mAU: milli-absorbance unit. Intensity AU: arbitrary unit.

H_2N-MKAKKWFESLFKTFFKKGKGIYPKSSFEKEKKTDKKKKFGGWVWFKK-$CONH_2$; peptide has been synthesized in 5 µmol scale. After purification (column 1, 05-50% MeCN), the 18 x TFA salt product (2.94 mg, 0.38 µmol, 8%) was obtained as a white solid. t_R = 11.35 min. Purity ≥ 99%. Formula: $C_{281}H_{428}N_{68}O_{61}S$. Molecular weight: 5766.88 g/mol. HRMS-ESI+ (m/z): $[M+6H]^{6+}$ calcd.: 962.0454; found: 962.0462.

.

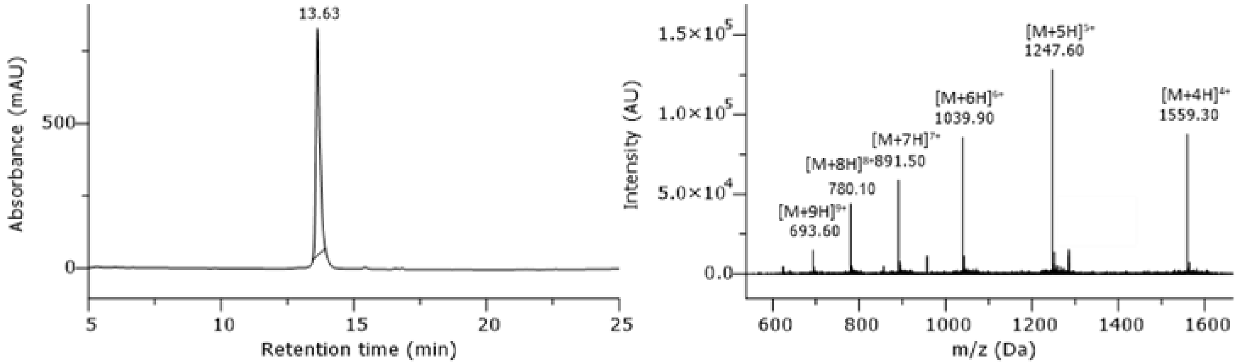

Supplementary Fig. 21: HPLC chromatogram of purified peptide AMP #17. Gradient 5-95% B in column 2, monitored at 220 nm. Absorbance mAU: milli-absorbance unit. Intensity AU: arbitrary unit.

H_2N-MWIKWKKPRKWGRRLKKKEKEELGDYIYLYCKVYRLFGFLPYFISKKTA-$CONH_2$; peptide has been synthesized in 5 µmol scale. After purification (column 1, 05-75% MeCN), the 16 x TFA salt product (2.73 mg, 0.34 µmol, 7 %) was obtained as a white solid. t_R = 13.63 min. Purity ≥ 99%. Formula: $C_{301}H_{462}N_{76}O_{64}S_2$. Molecular weight: 6233.48 HRMS-ESI+ (m/z): $[M+7H]^{7+}$ calcd.: 891.3618; found: 891.3609.

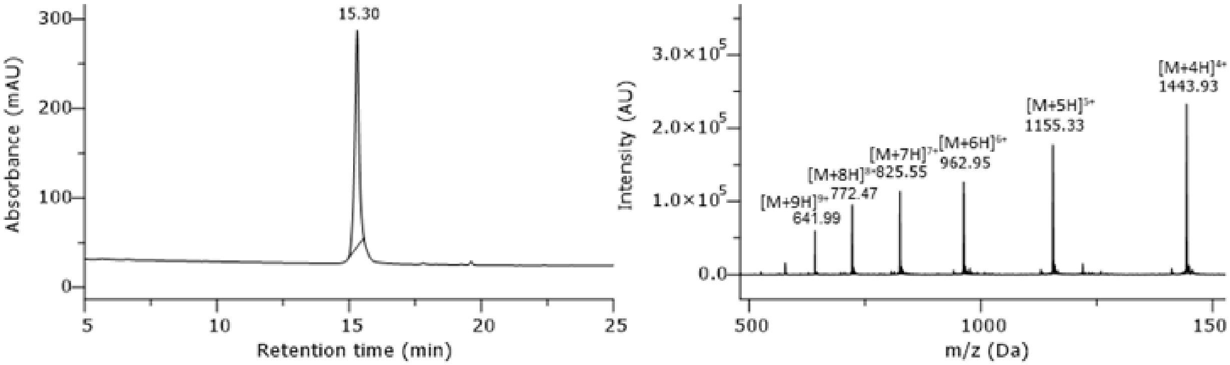

Supplementary Fig. 22: HPLC chromatogram of purified peptide AMP #18. Gradient 5-95% B in column 2, monitored at 220 nm. Absorbance mAU: milli-absorbance unit. Intensity AU: arbitrary unit.

H_2N-MCFFPRRKSKVKVKGGLCRLLFIFFKTTFCFKAKTKKEIKKGTGKKIVR-$CONH_2$; peptide has been synthesized in 5 µmol scale. After purification (column 1, 15-75% MeCN), the 17 x TFA salt product (2.99 mg, 0.39 µmol, 8%) was obtained as a white solid. t_R = 15.30 min. Purity ≥ 99%. Formula: $C_{270}H_{451}N_{75}O_{56}S_4$. Molecular weight: 5772.19 HRMS-ESI+ (m/z): $[M+10H]^{10+}$ calcd.: 578.1443; found: 578.1465.

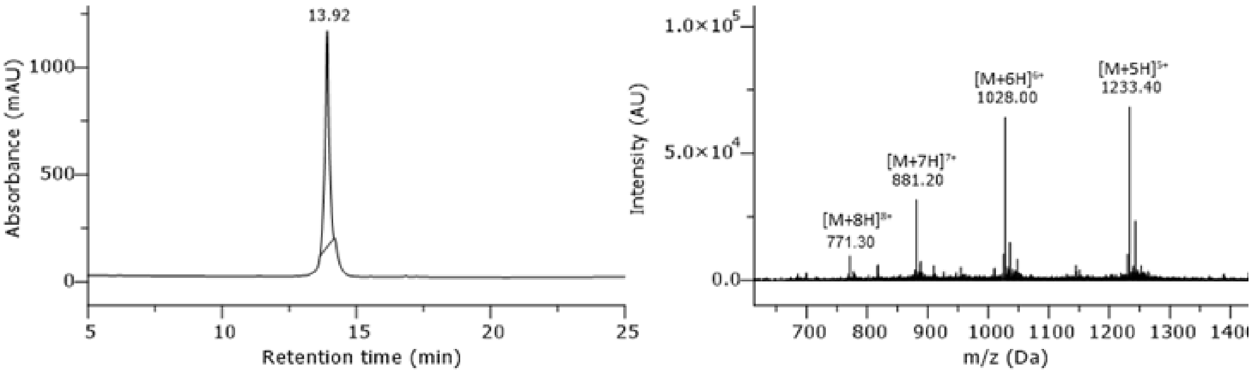

Supplementary Fig. 23: HPLC chromatogram of purified peptide AMP #19. Gradient 5-95% B in column 2, monitored at 220 nm. Absorbance mAU: milli-absorbance unit. Intensity AU: arbitrary unit.

H_2N-MCFCRYRFFYRRRIRFFKWGPYFYVWFGFPFGRKAFFLSVFRRRFC-$CONH_2$; peptide has been synthesized in 5 µmol scale. After purification (column 1, 05-75% MeCN), the 13 x TFA salt product (2.24 mg, 0.29 µmol, 6 %) was obtained as a white solid. t_R = 13.92 min. Approximate purity > 90%. Formula: $C_{305}H_{417}N_{81}O_{51}S_4$. Molecular weight: 6162.34 g/mol. HRMS-ESI+ (m/z): $[M+6H]^{6+}$ calcd.: 1028.0324; found: 1028.0393.

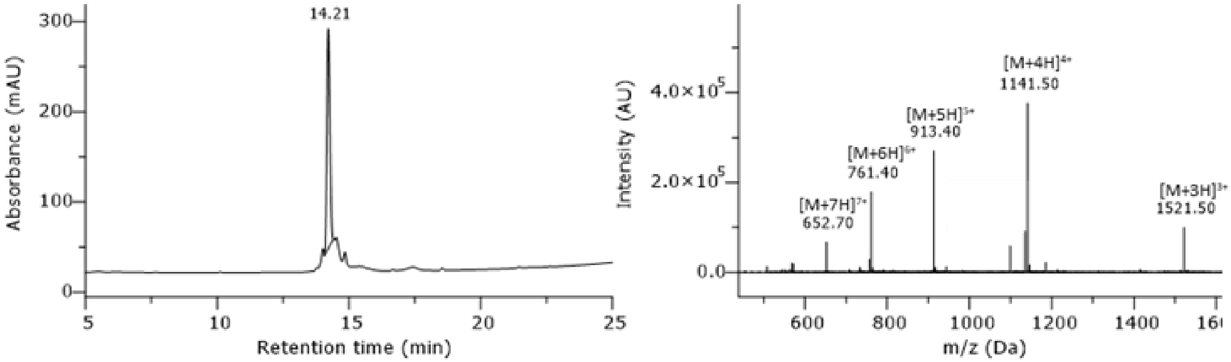

Supplementary Fig. 24: HPLC chromatogram of purified peptide AMP #20. Gradient 5-95% B in column 2, monitored at 220 nm. Absorbance mAU: milli-absorbance unit. Intensity AU: arbitrary unit.

H_2N-MRGRPPKRIRSVIIAQTTTATAKKIVIVLLLIFSSSKRRR-$CONH_2$; peptide has been synthesized in 5 µmol scale. After purification (column 1, 15-75% MeCN), the 12 x TFA salt product (0.35 mg, 0.06 µmol, 1 %) was obtained as a white solid. t_R = 14.21 min. Approximate purity > 85%. Formula: $C_{203}H_{367}N_{67}O_{49}S$. Molecular weight: 4562.57 g/mol. HRMS-ESI+ (m/z): $[M+5H]^{5+}$ calcd.: 913.3683; found: 913.3685.

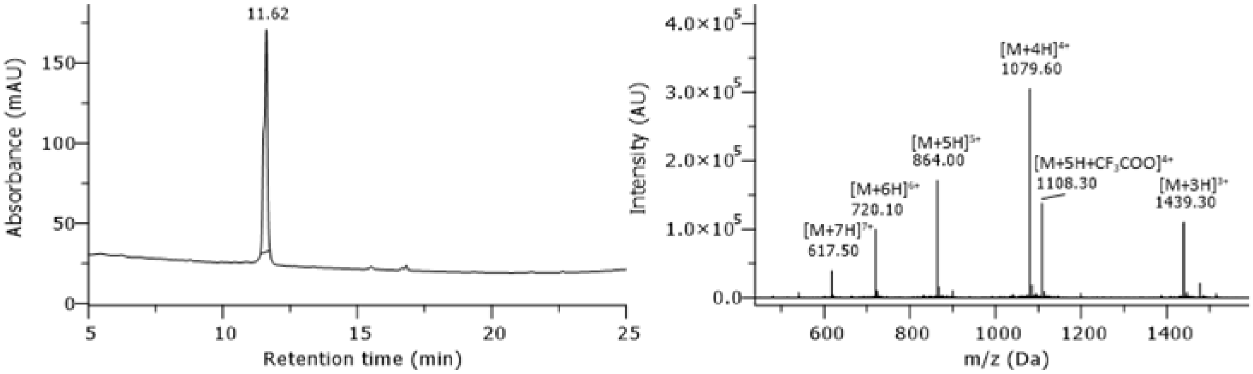

Supplementary Fig. 25: HPLC chromatogram of purified peptide AMP #21. Gradient 5-95% B in column 2, monitored at 220 nm. Absorbance mAU: milli-absorbance unit. Intensity AU: arbitrary unit.

H_2N-MGIGKFQKMRFIGAIRASKGVAKGLLRIAAIRTGRRALTT-$CONH_2$; peptide has been synthesized in 5 µmol scale. After purification (column 1, 05-50% MeCN), the 11 x TFA salt product (2.50 mg, 0.45 µmol, 9 %) was obtained as a white solid. t_R = 11.62 min. Approximate purity > 68%. Formula: $C_{191}H_{338}N_{64}O_{45}S_2$. Molecular weight: 4315.25 g/mol. HRMS-ESI+ (m/z): $[M+7H]^{7+}$ calcd.: 617.3733; found: 617.3767.

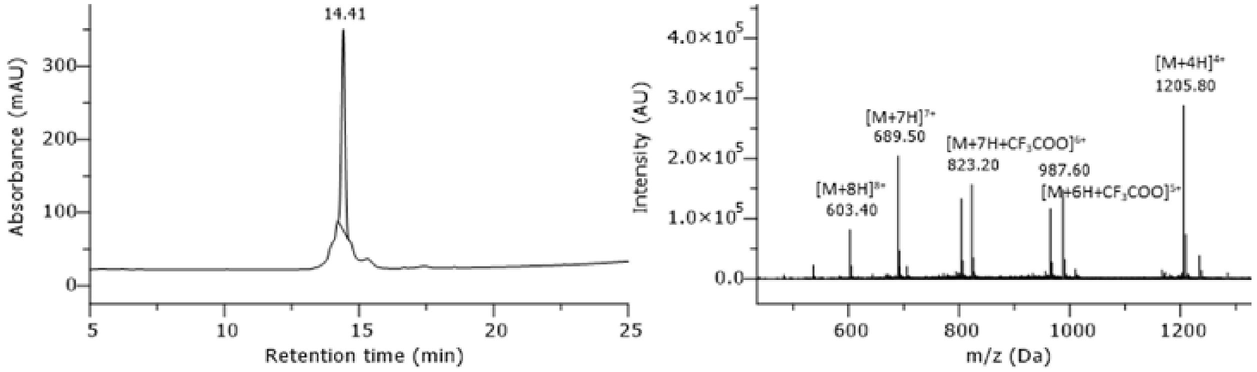

Supplementary Fig. 26: HPLC chromatogram of purified peptide AMP #23. Gradient 5-95% B in column 2, monitored at 220 nm. Absorbance mAU: milli-absorbance unit. Intensity AU: arbitrary unit.

H_2N-MSTRSSSIRRLVEAVRTRFRAALRTVLFFALRTTKRRPRR-$CONH_2$; peptide has been synthesized in 5 µmol scale. After purification (column 1, 15-75% MeCN), the 14 x TFA salt product (2.01 mg, 0.31 µmol, 6%) was obtained as a white solid. t_R = 14.41 min. Approximate purity 84%. Formula: $C_{209}H_{366}N_{78}O_{51}S$. Molecular weight: 4819.69 g/mol. HRMS-ESI+ (m/z): $[M+5H]^{5+}$ calcd.: 964.7716; found: 964.7743.

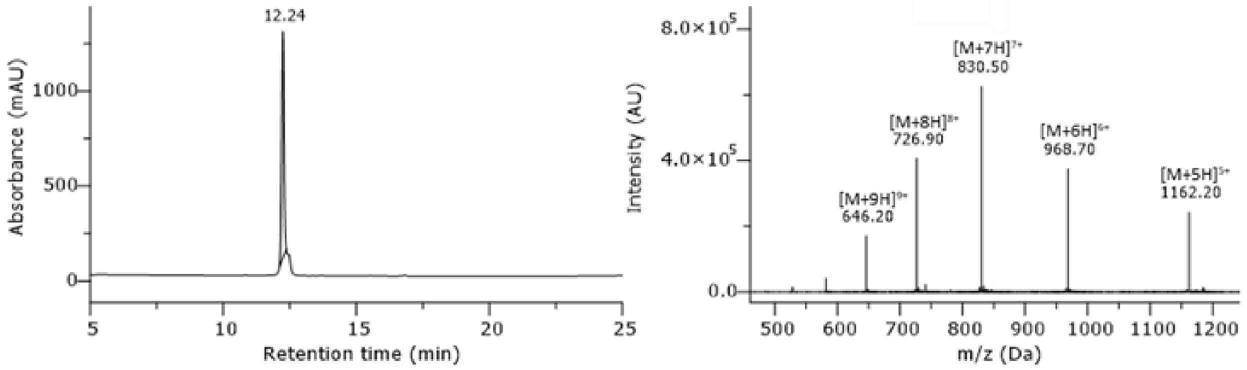

Supplementary Fig. 27: HPLC chromatogram of purified peptide AMP #24. Gradient 5-95% B in column 2, monitored at 220 nm. Absorbance mAU: milli-absorbance unit. Intensity AU: arbitrary unit.

H_2N-MAIRIIGRLARVRARVVARVRSRLLADDPPEDLLRVARRRKGRRWLFLS-$CONH_2$; peptide has been synthesized in 5 µmol scale. After purification (column 1, 05-50% MeCN), the 16 x TFA salt product (5.74 mg, 0.75 µmol, 15 %) was obtained as a white solid. t_R = 12.24 min. Approximate purity > 97%. Formula: $C_{255}H_{448}N_{94}O_{59}S$. Molecular weight: 5806.94 g/mol. HRMS-ESI+ (m/z): $[M+6H]^{6+}$ calcd.: 968.7530; found: 968.7544.

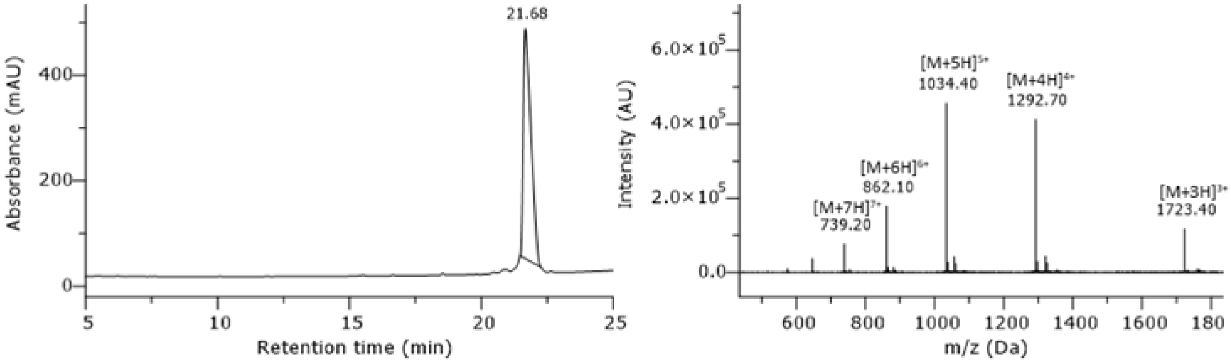

Supplementary Fig. 28: HPLC chromatogram of purified peptide AMP #26. Gradient 5-95% B in column 2, monitored at 220 nm. Absorbance mAU: milli-absorbance unit. Intensity AU: arbitrary unit.

H_2N-MKLRRRLRTRMVALLVLGVLFLLMLFFIIFLRRMLMRRFRA-$CONH_2$; peptide has been synthesized in 5 µmol scale. After purification (column 1, 20-95% MeCN), the 12 x TFA salt product (4.25 mg, 0.65 µmol, 13 %) was obtained as a white solid. t_R = 21.68 min. Purity 96%. Formula: $C_{241}H_{415}N_{73}O_{42}S_5$. Molecular weight: 5167.66 g/mol. HRMS-ESI+ (m/z): $[M+5H]^{5+}$ calcd.: 1034.4324; found: 1034.4339.

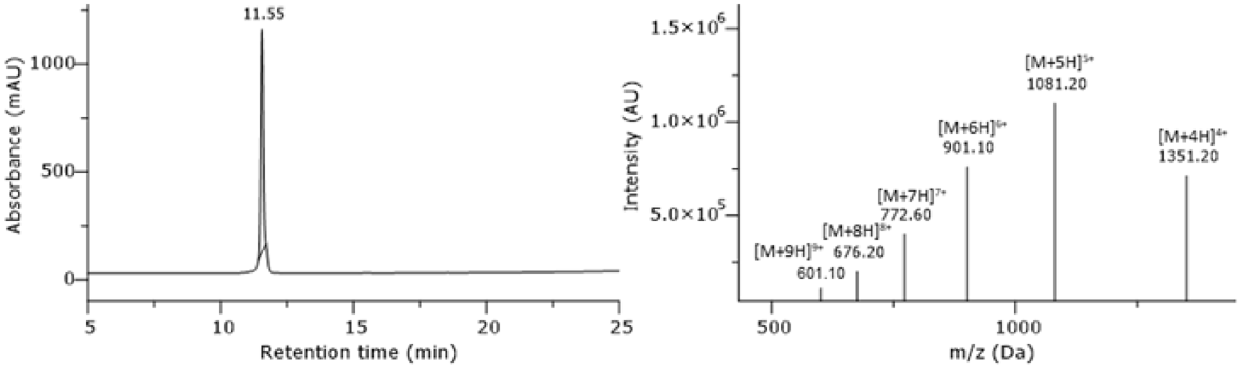

Supplementary Fig. 29: HPLC chromatogram of purified peptide AMP #27. Gradient 5-95% B in column 2, monitored at 220 nm. Absorbance mAU: milli-absorbance unit. Intensity AU: arbitrary unit.

H_2N-MNTTSNMIHRAVQQKRISFRAAKLTVLFLFKRRLLRRLLRHHEN-$CONH_2$; peptide has been synthesized in 5 µmol scale. After purification (column 1, 05-50% MeCN), the 15 x TFA salt product (4.03 mg, 0.57 µmol, 11%) was obtained as a white solid. t_R = 11.55 min. Purity ≥ 99%. Formula: $C_{239}H_{405}N_{83}O_{56}S_2$. Molecular weight: 5401.42 g/mol. HRMS-ESI+ (m/z): $[M+5H]^{5+}$ calcd.: 1081.225; found: 1081.2283.

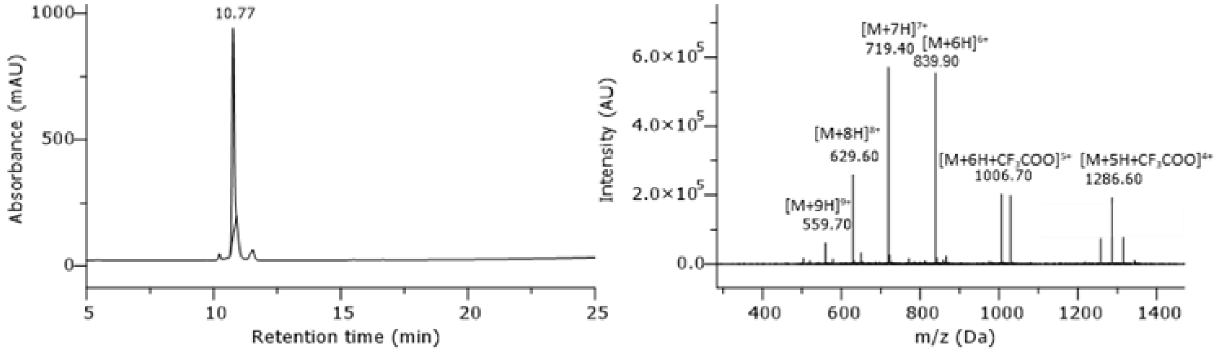

Supplementary Fig. 30: HPLC chromatogram of purified peptide AMP #28. Gradient 5-95% B in column 2, monitored at 220 nm. Absorbance mAU: milli-absorbance unit. Intensity AU: arbitrary unit.

H_2N-MMKIRNTLRSRKEAVRRIFSLRRRSVFTEMARAFRRFKAR-$CONH_2$; peptide has been synthesized in 5 µmol scale. After purification (column 1, 05-50% MeCN), the 16 x TFA salt product (4.53 mg, 0.66 µmol, 13 %) was obtained as a white solid. t_R = 10.77 min. Purity 90%. Formula: $C_{218}H_{377}N_{81}O_{50}S_3$. Molecular weight: 5029.03 g/mol. HRMS-ESI+ (m/z): $[M+7H]^{7+}$ calcd.: 719.4170; found: 719.4181.

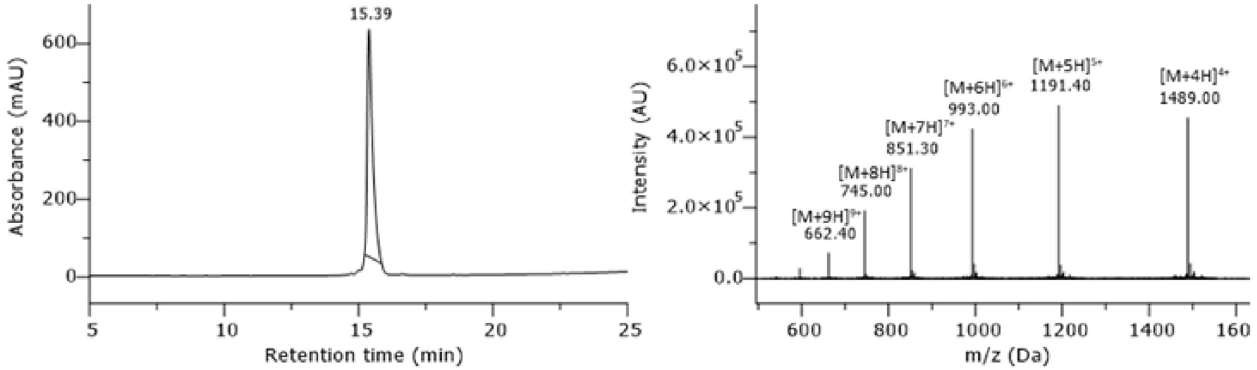

Supplementary Fig. 31: HPLC chromatogram of purified peptide AMP #29. Gradient 5-95% B in column 2, monitored at 220 nm. Absorbance mAU: milli-absorbance unit. Intensity AU: arbitrary unit.

H_2N-MKKKKKGILKQNNKKKKYTLFNRMVVFLFLGFIMIIFVQKYKKVIYHK-$CONH_2$; peptide has been synthesized in 5 µmol scale. After purification (column 1, 05-75% MeCN), the 17 x TFA salt product (4.80 mg, 0.61 µmol, 12 %) was obtained as a white solid. t_R = 15.39 min. Purity ≥ 99%. Formula: $C_{287}H_{471}N_{73}O_{57}S_3$. Molecular weight: 5952.45 g/mol. HRMS-ESI+ (m/z): $[M+5H]^{5+}$ calcd.: 1191.3161; found: 1191.3205.

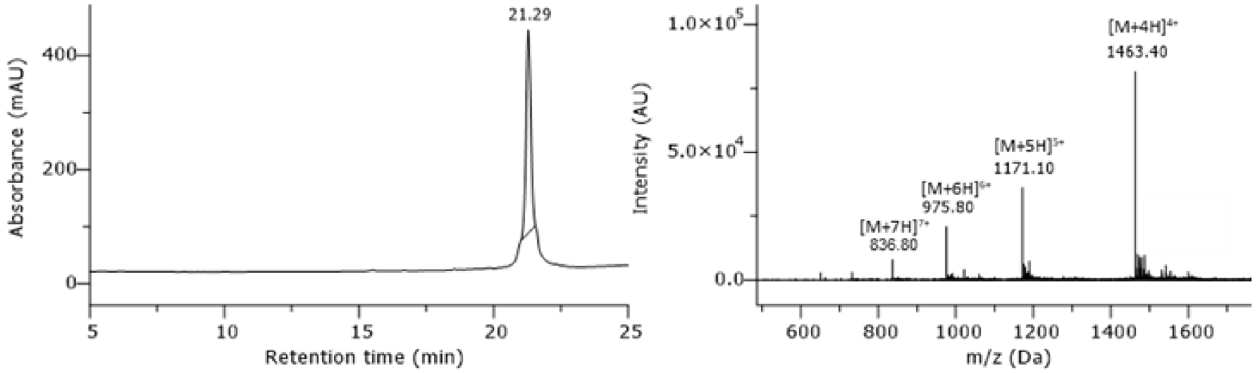

Supplementary Fig. 32: HPLC chromatogram of purified peptide AMP #30. Gradient 5-95% B in column 2, monitored at 220 nm. Absorbance mAU: milli-absorbance unit. Intensity AU: arbitrary unit.

H_2N-MGFGLWGLFHFKMNVPNLFKNGFIFLIIMIFTVWGLFFGKKKAYIEKFL-$CONH_2$; peptide has been synthesized in 5 µmol scale. After purification (column 1, 20-95% MeCN), the 8 x TFA salt product (1.44 mg, 0.21 µmol, 4 %) was obtained as a white solid. t_R = 21.29 min. Approximate purity 98%. Formula: $C_{295}H_{429}N_{63}O_{56}S_3$**.** Molecular weight: 5850.14 g/mol. HRMS-ESI+ (m/z): $[M+5H]^{5+}$ cacld.: 1170.8453; found: 1170.8442.

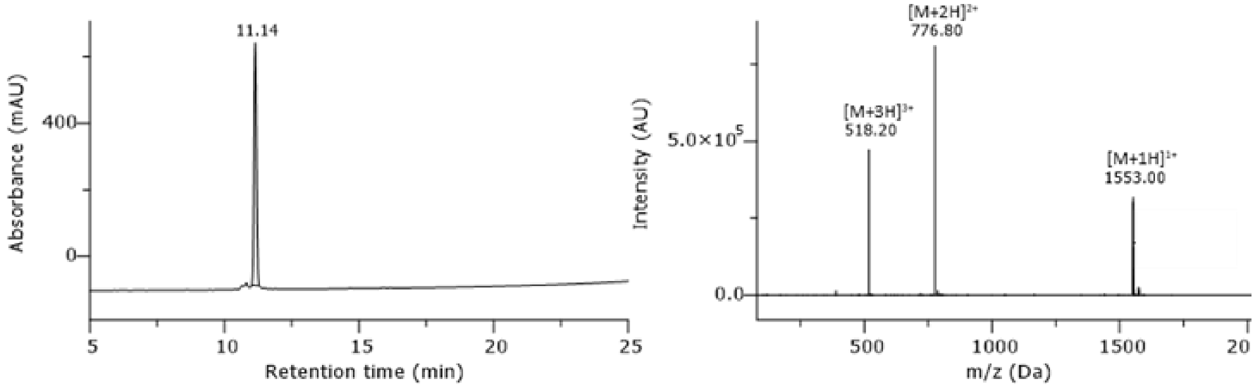

Supplementary Fig. 33 HPLC chromatogram of purified peptide BP100. Gradient 5-95% B in column 2, monitored at 220 nm. Absorbance mAU: milli-absorbance unit. Intensity AU: arbitrary unit.

H_2N-MKKLFKKILKYL-$CONH_2$; peptide has been synthesized in 5 µmol scale. After purification (column 1, 05-50% MeCN), the 6 x TFA salt product (4.03 mg, 1.80 µmol, 36 %) was obtained as a white solid. t_R = 11.14 min. Purity 97%. Formula: $C_{77}H_{134}N_{18}O_{13}S$. Molecular weight: 1552.06 g/mol. HRMS-ESI+ (m/z): $[M+2H]^{2+}$ calcd.: 776.5122; found: 776.5110.

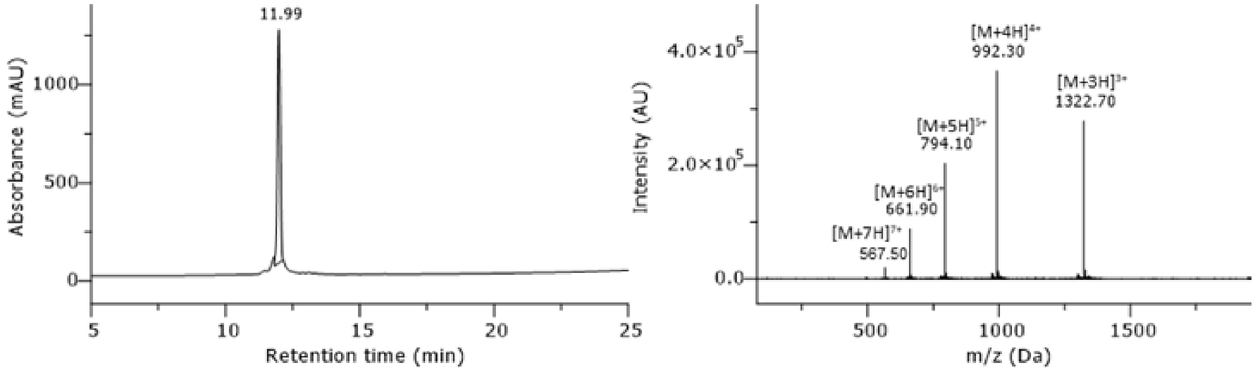

Supplementary Fig. 34: HPLC chromatogram of purified peptide Cecropin B. Gradient 5-95% B in column 2, monitored at 220 nm. Absorbance mAU: milli-absorbance unit. Intensity AU: arbitrary unit.

H_2N-MKWKVFKKIEKMGRNIRNGIVKAGPAIAVLGEAKAL-$CONH_2$; peptide has been synthesized in 5 µmol scale. After purification (column 1, 05-50% MeCN), the 10 x TFA salt product (2.29 mg, 0.45 µmol, 9%) was obtained as a white solid. t_R = 11.99 min. Purity 97%. Formula: $C_{181}H_{311}N_{53}O_{42}S_2$. Molecular weight: 3965.86 g/mol. HRMS-ESI+ (m/z): $[M+4H]^{4+}$ calcd.: 992.3404; found: 992.3409.

Supplementary references:

1. Espah Borujeni, A. & Salis, H. M. Translation initiation is controlled by RNA folding kinetics via a ribosome drafting mechanism. *J. Am. Chem. Soc.* **138**, 7016–7023 (2016).
2. Salis, H. M., Mirsky, E. A. & Voigt, C. A. Automated design of synthetic ribosome binding sites to control protein expression. *Nat. Biotechnol.* **27**, 946–950 (2009).
3. Das, P. *et al.* Accelerated antimicrobial discovery via deep generative models and molecular dynamics simulations. *Nature Biomedical Engineering* **5**, 613–623 (2021).
4. Jumper, J. *et al.* Highly accurate protein structure prediction with AlphaFold. *Nature* **596**, 583–589 (2021).
5. Tucs, A. *et al.* Generating Ampicillin-Level Antimicrobial Peptides with Activity-Aware Generative Adversarial Networks. *ACS Omega* **5**, 22847–22851 (2020).
6. Witten, J. & Witten, Z. Deep learning regression model for antimicrobial peptide design. *bioRxiv* (2019) doi:10.1101/692681.
7. Veltri, D., Kamath, U. & Shehu, A. Deep learning improves antimicrobial peptide recognition. *Bioinformatics* **34**, 2740–2747 (2018).
8. Meher, P. K., Sahu, T. K., Saini, V. & Rao, A. R. Predicting antimicrobial peptides with improved accuracy by incorporating the compositional, physico-chemical and structural features into Chou's general PseAAC. *Sci. Rep.* **7**, 42362 (2017).
9. Gawde, U. *et al.* CAMPR4: a database of natural and synthetic antimicrobial peptides. *Nucleic Acids Res.* **51**, D377–D383 (2023).
10. Pirtskhalava, M. *et al.* DBAASP v3: database of antimicrobial/cytotoxic activity and structure of peptides as a resource for development of new therapeutics. *Nucleic Acids Res.* **49**, D288–D297 (2021).
11. Flamm, C., Fontana, W., Hofacker, I. L. & Schuster, P. RNA folding at elementary step resolution. *RNA* **6**, 325–338 (2000).

12. Cock, P. J. A. *et al.* Biopython: freely available Python tools for computational molecular biology and bioinformatics. *Bioinformatics* **25**, 1422–1423 (2009).

13. Müller, A. T., Gabernet, G., Hiss, J. A. & Schneider, G. modlAMP: Python for antimicrobial peptides. *Bioinformatics* **33**, 2753–2755 (2017).
