# Supplementary material for: Cell-free biosynthesis combined with deep learning accelerates de novo-development of antimicrobial peptides
Source: Nat Commun. 2023 Nov 8;14:7197. doi: 10.1038/s41467-023-42434-9 (PMC10632401; doi:10.1038/s41467-023-42434-9)
Supplement: Supplementary file 3 — Reporting Summary [file 41467_2023_42434_MOESM3_ESM.pdf]

nature portfolio

Corresponding author(s): Tobias J. Erb & Amir Pandi

Last updated by author(s): Sep 20, 2023

Reporting Summary

Nature Portfolio wishes to improve the reproducibility of the work that we publish. This form provides structure for consistency and transparency in reporting. For further information on Nature Portfolio policies, see our Editorial Policies and the Editorial Policy Checklist.

Statistics

For all statistical analyses, confirm that the following items are present in the figure legend, table legend, main text, or Methods section.

n/a	Confirmed	
☐	☒	The exact sample size (n) for each experimental group/condition, given as a discrete number and unit of measurement
☐	☒	A statement on whether measurements were taken from distinct samples or whether the same sample was measured repeatedly
☐	☒	The statistical test(s) used AND whether they are one- or two-sided<br>*Only common tests should be described solely by name; describe more complex techniques in the Methods section.*
☒	☐	A description of all covariates tested
☒	☐	A description of any assumptions or corrections, such as tests of normality and adjustment for multiple comparisons
☐	☒	A full description of the statistical parameters including central tendency (e.g. means) or other basic estimates (e.g. regression coefficient) AND variation (e.g. standard deviation) or associated estimates of uncertainty (e.g. confidence intervals)
☐	☒	For null hypothesis testing, the test statistic (e.g. F, t, r) with confidence intervals, effect sizes, degrees of freedom and P value noted<br>*Give P values as exact values whenever suitable.*
☒	☐	For Bayesian analysis, information on the choice of priors and Markov chain Monte Carlo settings
☒	☐	For hierarchical and complex designs, identification of the appropriate level for tests and full reporting of outcomes
☒	☐	Estimates of effect sizes (e.g. Cohen's d, Pearson's r), indicating how they were calculated

Our web collection on statistics for biologists contains articles on many of the points above.

Software and code

Policy information about availability of computer code

Data collection	Bacterial growth data, PI fluorescence, and absorbance values were collected on Tecan Infinite 200 Pro plate reader with Magellan™ standard software. Outer membrane vesicles were collected using NanoAnalyzer (NanoFCM Co., Ltd, Nottingham, UK) with NanoFCM software (NF Profession V1.08). Images were collected using with a Zeiss AxioPlan 2 upright widefield microscope with MetaMorph version 6.2r6 software.
Data analysis	Data was analyzed using Python 3 scripts, MS Excel (2021), GraphPad Prism v9 and FiJi (version 1.54f).<br>All deep learning models were built, trained, and tested using Keras 1.0 with TensorFlow 2.0 backend using Python 3.9 in the Google Colab pro environment. The deep learning codes and models developed in this study as well as training data can be found at https://github.com/amirpandi/Deep_AMP. MD simulation setups can be found at 10.5281/zenodo.7327525.

For manuscripts utilizing custom algorithms or software that are central to the research but not yet described in published literature, software must be made available to editors and reviewers. We strongly encourage code deposition in a community repository (e.g. GitHub). See the Nature Portfolio guidelines for submitting code & software for further information.

Data

Policy information about availability of data

All manuscripts must include a data availability statement. This statement should provide the following information, where applicable:

- Accession codes, unique identifiers, or web links for publicly available datasets
- A description of any restrictions on data availability
- For clinical datasets or third party data, please ensure that the statement adheres to our policy

The training data used in this study along with the sequence of 500 tested AMPs, data underlying Supplementary Figs. 4 and 5 generated in this study can be found at https://github.com/amirpandi/Deep_AMP. Sequence of the 30 functional AMPs and source data underlying Fig. 4a generated in this study are provided in Supplementary Information (Supplementary Tables 5 and 10, respectively). The source data underlying Fig. 2b, Fig. 5a-d, Fig., Supplementary Fig. 1, Supplementary Fig. 2, and Supplementary Fig. 7 generated in this study are provided as Source Data files. Source data are provided with this paper.

Research involving human participants, their data, or biological material

Policy information about studies with human participants or human data. See also policy information about sex, gender (identity/presentation), and sexual orientation and race, ethnicity and racism.

Reporting on sex and gender	n/a, not applicable to this study. There was only a single healthy blood donor. We used the sample for in vitro cytotoxicity assay of antimicrobial peptides.
Reporting on race, ethnicity, or other socially relevant groupings	n/a
Population characteristics	n/a
Recruitment	The participant is one of the employees of the institute in which the experiment was performed (Bundeswehr Institute of Microbiology, Munich, Germany)
Ethics oversight	The study protocol is according to guidelines of Bundeswehr Institute of Microbiology, Munich, Germany, where the experiment was performed.

Note that full information on the approval of the study protocol must also be provided in the manuscript.

Field-specific reporting

Please select the one below that is the best fit for your research. If you are not sure, read the appropriate sections before making your selection.

☒ Life sciences ☐ Behavioural & social sciences ☐ Ecological, evolutionary & environmental sciences

For a reference copy of the document with all sections, see nature.com/documents/nr-reporting-summary-flat.pdf

Life sciences study design

All studies must disclose on these points even when the disclosure is negative.

Sample size	No sample size calculation was performed and sample size of 2-8 were chosen (depending on the type of experiments and availabilities according to common practice in the field) for experiments all specified throughout the manuscript and supplementary information (figure captions).
Data exclusions	No data was excluded from the analyses.
Replication	For experiments regarding screening and characterization of AMPs, biologically separate replicates were run simultaneously n=3 for Fig. 2b, Fig. 4a (MICs), Fig. 4b broad-band AMPs against Y. pestis and B. anthracis and S. pneumoniae, Fig. 5a, b (except for control, n=8), c, and n=2 for Fig. 4a (CC50), Fig. 4b ESKAPE pathogens. For HC50 experiment in Fig. 4a human blood was used in separate technical replicates (n=2). All attempts at replication were successful.<br><br>For cell-free experiments, n=3 replicates were run simultaneously by independently loading three separate reactions on the plate. All attempts at replication were successful.
Randomization	We did not have any group of participants and medical or environmental samples in this study to randomize to remove the bias. All sample used in this study were prepared by the investigation in the laboratory according to standards of the field, hence, no randomization was needed.
Blinding	In this study, blinding was not applicable because all samples, including proper controls, were prepared internally within the laboratory. There was no involvement of external individuals or subjective assessments, eliminating the need for blinding procedures

Reporting for specific materials, systems and methods

We require information from authors about some types of materials, experimental systems and methods used in many studies. Here, indicate whether each material, system or method listed is relevant to your study. If you are not sure if a list item applies to your research, read the appropriate section before selecting a response.

Materials & experimental systems

n/a	Involved in the study
☒	☐ Antibodies
☐	☒ Eukaryotic cell lines
☒	☐ Palaeontology and archaeology
☒	☐ Animals and other organisms
☒	☐ Clinical data
☒	☐ Dual use research of concern
☒	☐ Plants

Methods

n/a	Involved in the study
☒	☐ ChIP-seq
☒	☐ Flow cytometry
☒	☐ MRI-based neuroimaging

Eukaryotic cell lines

Policy information about cell lines and Sex and Gender in Research

Cell line source(s)	HCT 116 cell line (ATCC Biobank, CCL-247™) isolated cell line from the colon of an adult male with colon cancer.
Authentication	No authentication was performed
Mycoplasma contamination	The cell line was not tested for Mycoplasma contamination.
Commonly misidentified lines (See ICLAC register)	No commonly misidentified cell line was used in this study.
