# Supplementary material for: Cell-free biosynthesis combined with deep learning accelerates de novo-development of antimicrobial peptides
Source: Nat Commun. 2023 Nov 8;14:7197. doi: 10.1038/s41467-023-42434-9 (PMC10632401; doi:10.1038/s41467-023-42434-9)
Supplement: Supplementary file 4 — Source Data [file 41467_2023_42434_MOESM4_ESM.zip › Source Data/Raw images Source Data Fig. 5d.zip!/Raw images Source Data/Read me.pdf]

**First digit**	AMP
1	AMP3
2	AMP5
3	AMP13
4	AMP15
5	AMP16
6	AMP27
8	Cecropin B
10	No AMP

Second digit (1/2/3): biological replicate.
Third digit (1/2): different location of the slide.
PI: (propidium iodide) fluorescent.
PC: phase contract.
